# Metabolic predictors of COVID-19 mortality and severity: a survival analysis

**DOI:** 10.3389/fimmu.2024.1353903

**Published:** 2024-05-10

**Authors:** Abdallah Musa Abdallah, Asmma Doudin, Theeb Osama Sulaiman, Omar Jamil, Rida Arif, Fatima Al Sada, Hadi M. Yassine, Mohamed A. Elrayess, Abdel-Naser Elzouki, Mohamed M. Emara, Nagendra Babu Thillaiappan, Farhan S. Cyprian

**Affiliations:** ^1^ College of Medicine, Qatar University (QU) Health, Qatar University, Doha, Qatar; ^2^ Biomedical Research Center (BRC), Qatar University, Doha, Qatar; ^3^ Department of Medicine, Hamad General Hospital, Hamad Medical Corporation, Doha, Qatar; ^4^ Department of Radiology, Hamad General Hospital, Hamad Medical Corporation, Doha, Qatar; ^5^ Emergency Medicine Department, Hamad General Hospital, Hamad Medical Corporation, Doha, Qatar; ^6^ Neurosurgery Department, Hamad General Hospital, Hamad Medical Corporation, Doha, Qatar

**Keywords:** COVID-19, metabolites, biomarkers, severe, critical, mortality

## Abstract

**Introduction:**

The global healthcare burden of COVID-19 pandemic has been unprecedented with a high mortality. Metabolomics, a powerful technique, has been increasingly utilized to study the host response to infections and to understand the progression of multi-system disorders such as COVID-19. Analysis of the host metabolites in response to SARS-CoV-2 infection can provide a snapshot of the endogenous metabolic landscape of the host and its role in shaping the interaction with SARS-CoV-2. Disease severity and consequently the clinical outcomes may be associated with a metabolic imbalance related to amino acids, lipids, and energy-generating pathways. Hence, the host metabolome can help predict potential clinical risks and outcomes.

**Methods:**

In this prospective study, using a targeted metabolomics approach, we studied the metabolic signature in 154 COVID-19 patients (males=138, age range 48-69 yrs) and related it to disease severity and mortality. Blood plasma concentrations of metabolites were quantified through LC-MS using MxP Quant 500 kit, which has a coverage of 630 metabolites from 26 biochemical classes including distinct classes of lipids and small organic molecules. We then employed Kaplan-Meier survival analysis to investigate the correlation between various metabolic markers, disease severity and patient outcomes.

**Results:**

A comparison of survival outcomes between individuals with high levels of various metabolites (amino acids, tryptophan, kynurenine, serotonin, creatine, SDMA, ADMA, 1-MH and carnitine palmitoyltransferase 1 and 2 enzymes) and those with low levels revealed statistically significant differences in survival outcomes. We further used four key metabolic markers (tryptophan, kynurenine, asymmetric dimethylarginine, and 1-Methylhistidine) to develop a COVID-19 mortality risk model through the application of multiple machine-learning methods.

**Conclusions:**

Metabolomics analysis revealed distinct metabolic signatures among different severity groups, reflecting discernible alterations in amino acid levels and perturbations in tryptophan metabolism. Notably, critical patients exhibited higher levels of short chain acylcarnitines, concomitant with higher concentrations of SDMA, ADMA, and 1-MH in severe cases and non-survivors. Conversely, levels of 3-methylhistidine were lower in this context.

## 1 Introduction

The coronavirus disease 2019 (COVID-19) pandemic is an infectious disease caused by the severe acute respiratory syndrome coronavirus 2 (SARS-CoV-2). The global toll of COVID-19 has been unprecedented, which was first reported to the World Health Organization (WHO) on 31 December 2019, and has since caused a major global burden on healthcare, societies, and economies (1, 2). So far, more than 770 million confirmed COVID-19 cases and around 7 million deaths have been reported globally (3). COVID-19 mainly presents as a respiratory illness and the clinical spectrum ranges from asymptomatic or mild influenza-like illness to severe pneumonia with severe respiratory distress, which can lead to multi-organ dysfunction and failure, and death (4–6). An advanced age and pre-existing medical conditions such as hypertension, diabetes, obesity, and smoking amongst others, have been linked to adverse clinical outcomes in COVID-19 (7–9). Indeed, patients with such characteristics will be at a higher risk of developing a serious illness with severe or life-threatening consequences (6). There has been a massive and unified global effort to enhance our understanding of the disease and the interaction between the pathogen and the human host, which has resulted in successful production and implementation of a vaccination strategy to control the spread of this disease (10, 11). However, perturbation of multiple physiological pathways in humans by SARS-CoV-2 and the resultant complexities in clinical presentations, make it challenging to arrive at an accurate patient risk stratification. Higher incidences of adverse clinical outcomes in COVID-19 patients with comorbidities, such as older age, diabetes, dyslipidemia and obesity suggest that metabolic disturbances might play key roles in COVID-19 severity and outcomes (12, 13). Therefore, to improve clinical management, there is a need to better understand the impact of COVID-19 on host metabolic profile, which might underlie the differences in clinical presentation.

Metabolomics is a powerful technique, which is gaining much traction as potential diagnostic, monitoring and prognostic tool. It allows quantitative analyses of large datasets of biomolecules (also known as metabolites) from host biological specimen, which can provide a broad picture of the metabolome and insights into complex metabolic pathways. This metabolic profile can act as a snapshot of the patient’s metabolome, providing a detailed description of the metabolic state as a result of both genetic contributions and environmental factors (14). Metabolomics can also allow examination of disease-induced changes to the host metabolic landscape and thereby help identify biomarkers of disease severity, predict patient outcomes and facilitate therapeutic intervention (12, 15).

Metabolomics has been leveraged in biomarker discovery to identify metabolites correlating with diseases. Multiple studies have reported metabolic dysregulation during COVID-19 progression (16–24) and their effect on multiple organ systems (19), which suggest that these metabolites may be used as prognostic markers. For example, increases in the ratios of kynurenine to tryptophan (25, 26), and arginine to ornithine (26, 27) and a decrease in the ratio glutamine to glutamate (25, 28, 29) have been reported in COVID-19 patients suggestive of COVID-induced metabolic changes. In addition, triglycerides, were also found to be upregulated in COVID-19 patients and it positively correlates with pro-inflammatory markers such as interleukin-6 (IL-6) and C-reactive protein (CRP) (16, 23, 26, 30). Altogether, these studies have identified alterations in amino acids, lipids, and other crucial metabolic pathways due to COVID-19, however, more research is necessary to characterize and validate biomarkers that can predict the course of the disease, with the ultimate goal of reducing critical complications. In this study, we report the metabolomic changes associated with COVID-19 infection during the early phase of the pandemic from July 2020 to October 2020 and link them to survival probability of COVID-19 patients in Qatar. We aim to stratify patient risk and disease progress based on the metabolic profile and identify biomarkers that are robust in diagnosis and prognosis of COVID-19.

## 2 Materials and methods

### 2.1 Study participants

This prospective cohort study included 154 adult patients diagnosed with COVID-19 at Hamad Medical Corporation from July to December 2020. The participants were mostly males (n=138), with a mean age of 55 (range: 48–69) and diverse nationalities. Based on the WHO classification of clinical presentation, patients were divided into five age matched groups: asymptomatic (n=36), mild symptomatic (n=23), mild pneumonia (n=32), severe (n=23), and critical (n=40) (31). Around 10 ml venous blood samples was collected either at the time of diagnosis or hospital admission from all consenting adults above 18 years of age. The plasma was aliquoted after centrifuging the blood samples at 3000 rpm for 5 min at 4°C, and stored in -80°C. The available clinical and laboratory data, such as body mass index (BMI), viral load, and blood test results, were obtained from the hospital’s electronic healthcare system with patients’ consent. The study was approved by the institutional review boards of Hamad Medical Corporation (MRC-01-20-145) and Qatar University (QU-IRB 1289-EA/20).

### 2.2 Metabolomics

The targeted metabolomics of serum samples collected from all participants within 24 to 48 hours after diagnosis was performed using Biocrates MxP^®^ Quant 500 Kit (Biocrates, Innsbruck, Austria). Tandem mass spectrometry was performed at the Fraunhofer Institute for Toxicology and Experimental Medicine. We analyzed 630 metabolites as part of the MetIDQ™ MetaboINDICATOR™ module designed for MxP^®^ Quant 500 kit. Lipid quantification was performed using Flow Injection Analysis Tandem Mass Spectrometry (FIA-MS/MS), and small molecule quantification was done using liquid chromatography-tandem mass spectrometry (LC-MS/MS) with the 5500 QTRAP^®^ instrument triple quadrupole mass spectrometer (AB Sciex, Darmstadt, Germany) as previously described (32, 33).

### 2.3 Statistical analysis

All analyses were performed using R version 3.6.3 and python. Data reprocessing was carried out, including normalization by median, log transformation, and Pareto scaling, before clustering to construct the heat-maps. Principal Component Analysis (PCA) was performed to examine the metabolic profiles of COVID-19 patients, with the goal of identifying factors associated with severity and survival. Wilcoxon rank sum tests were implemented to determine the significance of differences between different severity and survival groups. Statistical differences between groups were considered statistically significant if the p-value was less than 0.05. In addition, Receiver Operating Characteristic (ROC) curve analysis was utilized to evaluate the predictive capability of certain metabolites in determining survival status in our cohort. Correlation between certain metabolites and clinical markers was analyzed using the Spearman correlation method. Using Kaplan Meier survival analysis, significant indicators of patient survival related to different metabolite levels were identified, while the Youden method was employed to determine the optimal cut-points for the variables. Survival time was defined as the time from hospital admission to discharge or death. Volcano plots and heat-maps were generated using the Metaboanalyst R package to compare metabolomic differences between patients across different severity groups.

The dataset utilized in this study for creation and testing of models comprises 154 samples, each representing a unique patient record. The dataset summarized in **Table 1**, provides information on demographics, including diabetes and hypertension status as well as age, gender, and body mass index (BMI) (n=154). In this study, various machine learning (ML) algorithms were employed to analyze data with the aim of predicting living status based on metabolic measurements. From the dataset, we tested specific metabolites as predictors for our models, including Tryptophan, Kynurenine, Asymmetric dimethylarginine, and 1-Methylhistidine. These predictors were chosen based on their potential relevance to the living status outcome, literature review and univariate analysis. The dataset of 154 samples was randomly divided into training and testing sets, with 80% of the data used for training and 20% for testing. To enhance reproducibility, we incorporated a random seed using “random.seed()” function in our code to ensure that data splitting and model initialization, or random processes, yield consistent results across different runs. The ML models, including Logistic Regression, Random Forest Classifier, Support Vector Machine (SVM), Bernoulli Naive Bayes, Gradient Boosting Classifier (using XGBoost), K-Nearest Neighbors (KNN), Neural Network (Multilayer Perceptron), and MLPClassifier were trained using the training set and then used to make predictions on the testing set. The performance of each model was evaluated based on its accuracy and confusion matrix, including the true positives, false positives, true negatives, and false negatives predicted by the model. The python packages used were pandas and scikit-learn (all the codes are available as **Supplementary Material Datasheet 3**).

**Table 1:** Clinical traits of participants stratified by asymptomatic, mild symptomatic, mild pneumonia, severe and critical COVID-19 cases.

			Severity					
Characteristic	N	Total	Asymptomatic N = 36^1^	Mild Symptomatic N = 23^1^	Mild Pneumonia N = 32^1^	Severe N = 23^1^	Critical N = 40^1^	p-value^2^
**Living status**	154							**<0.001**
Non-survivor		33 (100%)	0 (0%)	0 (0%)	1 (3.0%)	5 (15%)	27 (82%)	
Survivor		121 (100%)	36 (30%)	23 (19%)	31 (26%)	18 (15%)	13 (11%)	
**Gender**	154							**0.011**
Female		16 (100%)	1 (6.2%)	1 (6.2%)	9 (56%)	1 (6.2%)	4 (25%)	
Male		138 (100%)	35 (25%)	22 (16%)	23 (17%)	22 (16%)	36 (26%)	
**Age**	154	55 (48-63)	52 (46-55)	52 (46-56)	51 (47-61)	60 (54-65)	64 (56-73)	**<0.001**
**Diabetes mellitus**	154	77 (100%)	12 (16%)	12 (16%)	18 (23%)	12 (16%)	23 (30%)	0.2
**Hypertension**	154	79 (100%)	14 (18%)	7 (8.9%)	18 (23%)	15 (19%)	25 (32%)	**0.035**
**White blood cell count (WBC) [x10^3^/uL]**	152	7.5 (5.6-11.5)	6.3 (5.3-7.3)	6.2 (5.0-8.8)	6.1 (4.4-8.3)	9.0 (7.1-12.4)	12.6 (8.7-15.8)	**<0.001**
**Red blood cell count (RBC) [x10^6^/uL]**	152	4.60 (3.60-5.12)	5.10 (4.85-5.40)	5.25 (4.93-5.77)	4.90 (4.50-5.23)	4.00 (3.50-4.50)	3.10 (2.80-3.82)	**<0.001**
**Hemoglobin (Hgb) [g/dL]**	152	12.55 (10.47-14.60)	14.60 (13.55-15.35)	14.80 (13.65-16.08)	13.40 (12.38-14.17)	11.90 (10.85-12.50)	9.15 (8.10-11.05)	**<0.001**
**Hematocrit (Hct) [%]**	152	38 (32-43)	44 (41-45)	44 (41-48)	40 (38-43)	35 (32-37)	28 (25-33)	**<0.001**
**Mean corpuscular volume (MCV) [fL]**	152	86 (82-90)	86 (82-89)	83 (80-86)	84 (76-88)	89 (85-93)	89 (87-92)	**<0.001**
**Absolute neutrophil count (ANC) [x10^3^/uL]**	152	5.3 (3.1-8.9)	3.7 (2.5-4.7)	3.5 (2.1-4.3)	3.7 (2.5-5.8)	7.3 (5.7-10.7)	10.4 (7.5-13.8)	**<0.001**
**Lymphocyte count [x10^3^/uL]**	152	1.40 (0.98-2.00)	1.70 (1.55-2.45)	2.00 (1.60-2.40)	1.35 (1.08-1.83)	1.10 (0.65-1.50)	0.90 (0.50-1.40)	**<0.001**
**Mean platelet volume (MPV) [fl]**	147	10.50 (9.90-11.50)	10.10 (9.55-10.95)	10.45 (9.70-11.30)	10.40 (10.05-10.95)	10.40 (9.60-11.45)	11.30 (10.38-12.33)	**<0.001**
**Red blood cell distribution width (RDW-CV) [%]**	152	13.70 (12.60-16.00)	12.30 (11.90-12.95)	12.80 (12.25-14.00)	13.50 (12.67-14.62)	14.30 (13.65-15.55)	16.65 (14.60-19.52)	**<0.001**
**D-Dimer [mg/L FEU]**	103	1.35 (0.54-3.77)	0.28 (0.24-0.61)	0.53 (0.33-1.08)	0.48 (0.36-0.69)	1.87 (0.84-3.28)	3.35 (1.78-5.29)	**<0.001**
**Total Protein [g/L]**	132	70 (64-76)	77 (72-82)	73 (72-75)	69 (66-72)	70 (64-78)	64 (57-72)	**<0.001**
**Albumin [g/L]**	144	31 (25-38)	40 (38-44)	39 (36-42)	32 (30-35)	26 (24-30)	23 (20-27)	**<0.001**
**Alkaline phosphatase (ALP) [U/L]**	138	92 (70-129)	82 (70-104)	63 (60-74)	78 (64-94)	108 (85-148)	150 (96-287)	**<0.001**
**Calcium [mmol/L]**	143	2.22 (2.12-2.33)	2.32 (2.28-2.38)	2.33 (2.23-2.40)	2.18 (2.14-2.31)	2.14 (2.08-2.28)	2.12 (2.05-2.20)	**<0.001**
**C-reactive protein (CRP) [mg/L]**	152	18 (5-69)	4 (2-11)	3 (2-9)	41 (17-83)	40 (10-101)	67 (40-111)	**<0.001**
**Ferritin [ug/L]**	104	688 (318-1,371)	218 (144-396)	265 (114-365)	518 (219-860)	670 (438-1,124)	1,276 (704-2,656)	**<0.001**

Parametric traits are described with mean ± sd, non-parametric using median, whilst categorical variables are given in counts. Significant p-values are in bold text.

^1^n (%); Median (25%-75%).

^2^Fisher’s exact test; Kruskal-Wallis rank sum test; Pearson’s Chi-squared test.

## 3 Results

### 3.1 Characterization of study patients

The study population consisted of 154 SARS-CoV-2 RT-PCR positive COVID-19 cases that presented with asymptomatic, mild symptomatic, mild pneumonia, severe and critical clinical phenotypes. Baseline demographic and clinical characteristics (clinical data with cutoffs across the five severity groups of COVID-19 patients) are summarized in **Table 1**. Cohorts were matched for age and 121 (121/154, 79%) of enrolled patients survived the infection (**Table 1**). Prevalence of diabetes and hypertension were higher in critical group (**Table 1**). Inflammatory biomarkers, such as IL-6 and CRP, are predictive biomarkers in COVID-19 patients (34) and were significantly increased in the COVID-19-positive groups. **Table 1** summarizes the clinical and laboratory characteristics of the enrolled cohort. Thromboembolic complications have been reported commonly in severe COVID-19 infections. Levels of D-dimer and CRP were significantly elevated in COVID-19 patients, which correlated with severity of COVID-19 symptoms. Our study revealed a positive correlation between serum ferritin levels and disease severity, poor prognosis and mortality, suggesting that ferritin levels could be an indicator of disease severity and clinical outcome (**Table 1**). Increased levels of ferritin in severe disease might indicate an underlying dysregulation in iron metabolism in response to COVID-19 infection. Therefore, monitoring serum ferritin levels can serve as an important predictive biomarker in COVID-19 management. In addition, the decline in serum albumin levels correlated with disease severity and mortality in our COVID-19 cohort. Moreover, our study also revealed that COVID-19 patients with a severe disease and those that did not survive, had higher serum triglycerides compared to those with less severe disease.

### 3.2 Stratification of COVID-19 clinical phenotypes using metabolomics

**Figure 1:**
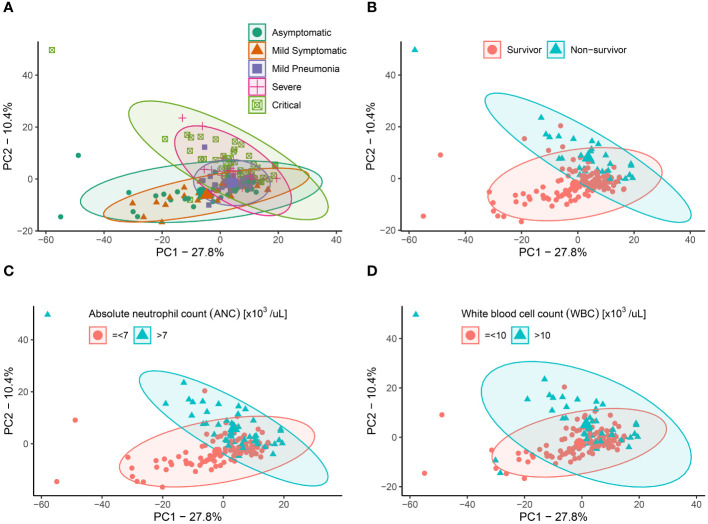
Principal components analysis (PCA) for COVID-19 patients based on putative metabolites. **(A)** PCA plot showing the distribution of 609 metabolites in individuals classified as Asymptomatic (green), Mild Symptomatic (orange), Mild Pneumonia (purple), Severe (pink), and Critical (green) based on their clinical symptoms. **(B)** PCA plot showing the distribution of 609 metabolites in individuals classified as survivors (blue) and non-survivors (pink) of COVID-19. **(C)** PCA plot showing the neutrophil counts in COVID-19 patients with asymptomatic/mild (ANC=<7) and severe/critical (ANC>7) groups. **(D)** PCA plot showing the WBC counts in COVID-19 patients with =<10 for asymptomatic/mild and >10 for severe/critical groups.

In the current study, we employed Kaplan-Meier survival analysis to evaluate the correlation between metabolic markers that were previously reported and patient outcomes. A hierarchical cluster analysis of identified metabolites revealed that COVID-19 symptoms severity clearly differed in their metabolic signatures, indicating that the observed metabolic alteration is indeed specific to COVID-19 patients (**Supplementary Figure 1**). 609 metabolites were analyzed in patients classified as asymptomatic (n=36), mild symptomatic (n=23), mild pneumonia (n=32), severe (n=23) and critical (n=40) based on WHO classification. Distinct metabolites allowed for discrimination of COVID-19 clinical symptoms (**Supplementary Figure 1**) and this was further highlighted by the scaled principal component analysis (PCA), which revealed metabolic phenotypes of sera from COVID-19 asymptomatic/mild symptomatic groups differing substantially from severe/critical groups (**Figure 1A**). We examined the relationship between survival and metabolic profile of COVID-19 patients and a PCA plot revealed a clear separation between survivors and non-survivors (**Figure 1B**). Similarly, a PCA plot of neutrophil counts in COVID-19 patients revealed similar distributions as in patients with asymptomatic/mild (ANC=<7) and severe/critical (ANC>7) groups, indicating that a severe disease positively correlates with a high neutrophil count (**Figure 1C**). An analogous PCA plot of WBC counts (=<10 for asymptomatic/mild and >10 for severe/critical groups) revealed similar results (**Figure 1D**). The observed clear separations between the COVID-19 clinical phenotypes for neutrophil and WBC counts indicates a potential association between the metabolic changes in these subgroups and disease severity. Neutrophils rely on both the tricarboxylic acid (TCA) cycle and the pentose phosphate pathway (PPP) to achieve their desired outcomes as effector cells by making reactive oxygen species (ROS) (35). However, when crucial amino acids like arginine and histidine are depleted, it can severely impair the functionality of neutrophils even with high neutrophil count (36). On the other hand, the proliferation of lymphocytes is contingent upon the availability of tryptophan (37). Volcano plots highlighted the most differentially expressed metabolites in COVID-19 plasma samples associated with disease severity distinct metabolites in COVID-19 plasma samples compared to controls (**Supplementary Figure 1**). A comparison of survival rates between individuals presenting with elevated levels of several metabolites, and those with lower levels, revealed statistically significant differences in survival outcomes. These metabolites included amino acids, tryptophan and kynurenine, their associated metabolites, creatine, symmetric dimethylarginine (SDMA), asymmetric dimethylarginine (ADMA), 1-methylhistidine (1-MH), as well as carnitine palmitoyltransferase 1 and 2 enzymes indicators (**Supplementary Figures 2**, **3**).

### 3.3 Association between circulating amino acids profile and COVID-19 severity

**Figure 2:**
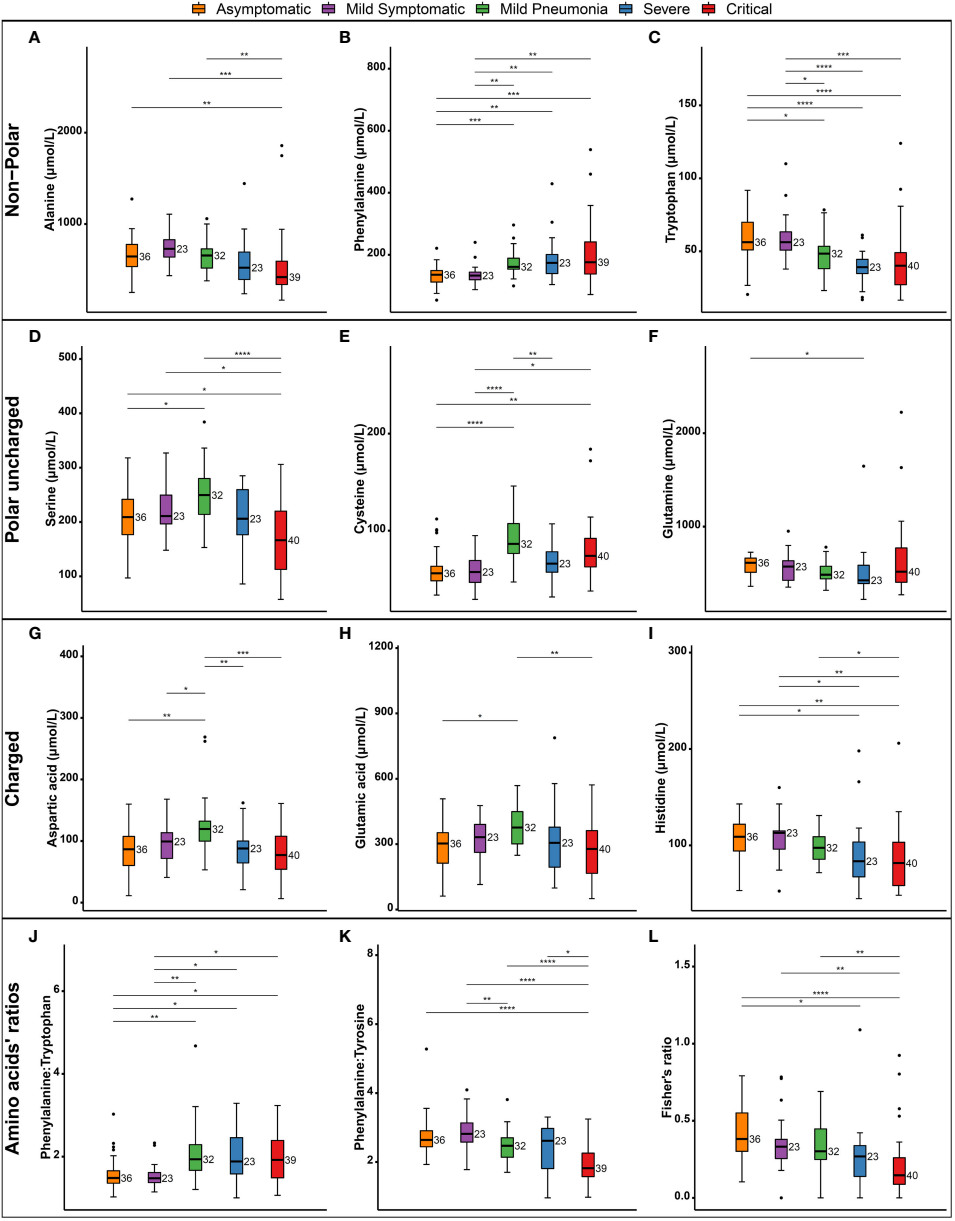
Changes in circulating amino acids associated with COVID-19 clinical severity. Box and whisker plots showing the levels of various amino acids in patients classified by severity of illness. Non-polar amino acids **(A–C)**, polar uncharged amino acids **(D–F)**, Charged amino acids **(G–I)**, ratios of phenylalanine to tryptophan **(J)** and to Tyrosine **(K)** are shown. Fishers ratio is depicted **(L)**. The boxes depict the interquartile range (IQR) and the whiskers extend to the most extreme data points that are not outliers. Outliers are indicated by black circles. The following symbols were used to indicate statistical significance in differences in the levels of amino acids between different severity groups NS, *(0.05); **(0.01); ***(0.001); ****(0.0001).

**Figure 3:**
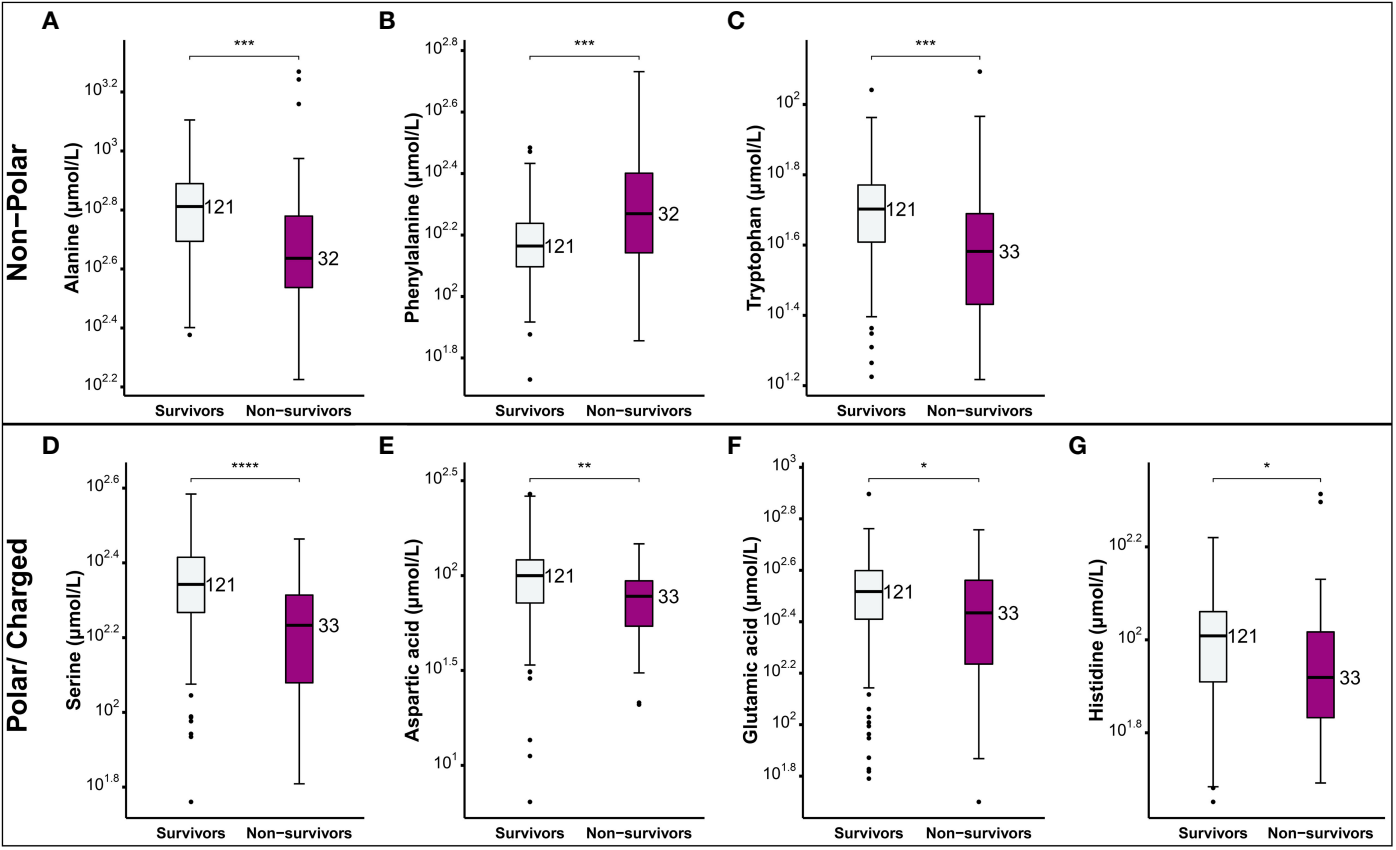
Comparison of the levels of amino acids between COVID-19 patients who survived and those who did not. Non-polar amino acids **(A-C)** and polar charged amino acids **(D-G)**. Box plots depict the interquartile range (IQR) and the whiskers extend to the most extreme data points that are not outliers. Outliers are indicated by black circles. The following symbols were used to indicate statistical significance in differences of the levels of amino acids between survivors and non-survivors NS, *(0.05); **(0.01); ***(0.001); ****(0.0001).

The severity spectrum of COVID-19 symptoms has been associated with circulating amino acids concentrations (38). Hence, we examined the correlation between the amino-acid profile and the severity of the disease in COVID-19 patients. We measured the levels of alanine, phenylalanine, tryptophan, serine, cysteine, glutamine, aspartic acid, glutamic acid, and histidine in different clinical phenotypes (**Figure 2**). We found that levels of certain amino acids, such as alanine, tryptophan, serine, glutamine, and histidine were significantly reduced in critical and severe cases of COVID-19 and this reduction was associated with disease severity (**Figure 2**). On the other hand, levels of phenylalanine and cysteine were increased in severe and critical patients compared to asymptomatic group (**Figures 2B, E**). Interestingly, levels of some amino acids, such as serine (**Figure 2D**) and aspartic acid (**Figure 2G**) varied among different severity groups. While aspartic acid levels were lowest in critically-ill patients, its levels were highest in those with mild-pneumonia (**Figure 2G**). These findings suggest that changes to amino acid profile can be associated with severity of COVID-19 symptoms. Moreover, in severe COVID-19 patients, we found an increase in the ratio of phenylalanine to tryptophan (**Figure 2J**) (suggestive of decreased protein synthesis), as well as a decrease in the ratio of phenylalanine to tyrosine (**Figure 2K**) (suggesting the utilization to generate neurotransmitters) in comparison to mild and asymptomatic patients. Consistent with other studies, Fisher’s ratio correlated negatively with the disease severity (39) (**Figure 2L**). Similarly, analysis of serum amino acid levels revealed significant differences between survivors and non-survivors (**Figure 3**). The levels of alanine, tryptophan, serine, aspartic acid, glutamic acid, and histidine were found to be significantly decreased in individuals who did not survive in comparison to those who did. On the other hand, the levels of phenylalanine were found to be elevated in non-survivors (**Figure 3**). This data correlates well with the amino acid levels observed in severe/critical cases (**Figure 2**).

### 3.4 Alterations in tryptophan and kynurenine metabolism and kynurenine/tryptophan ratio

**Figure 4:**
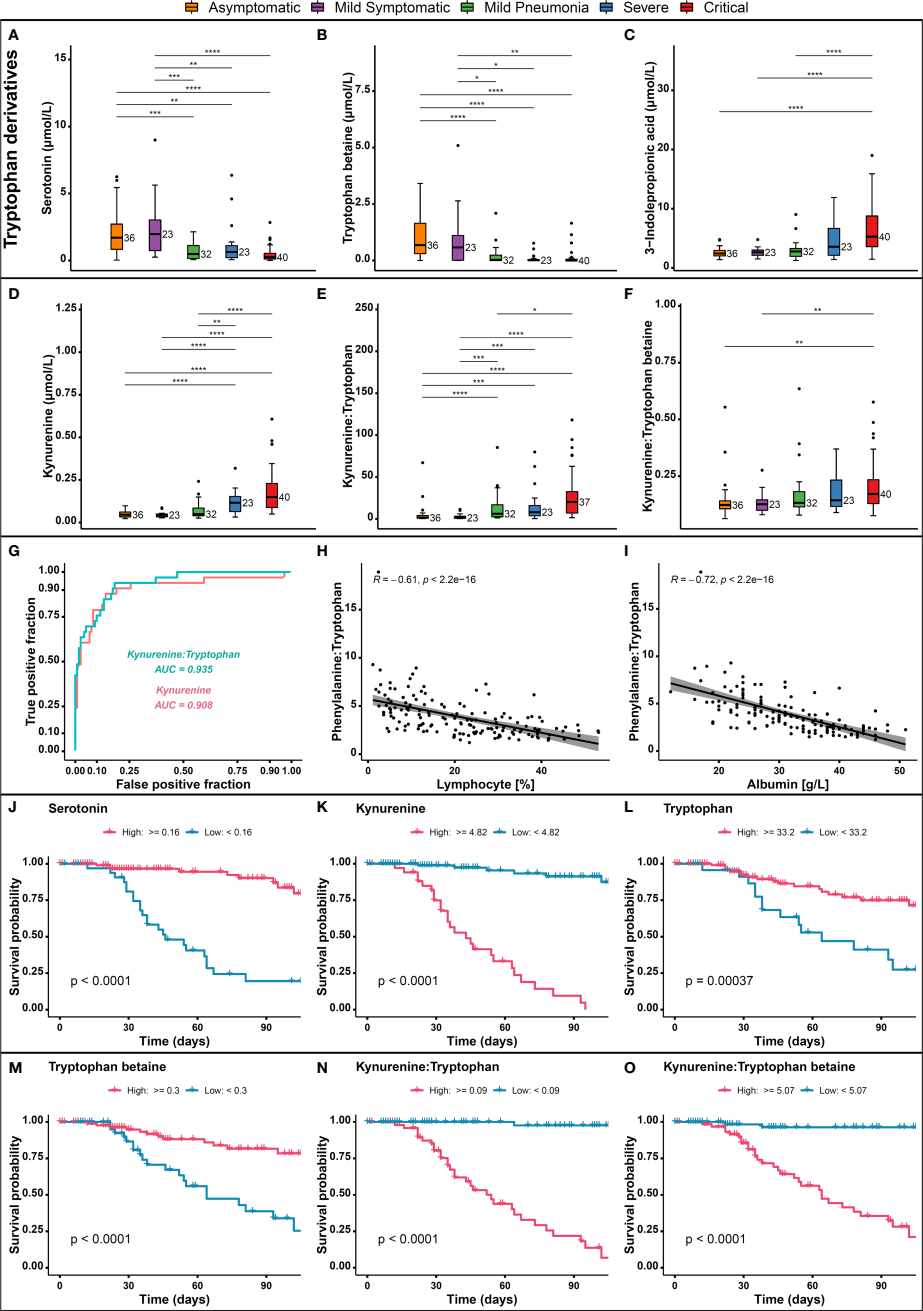
Alterations in tryptophan and kynurenine pathway metabolites. Levels of **(A)** Serotonin, **(B)** Tryptophan betaine, **(C)** 3-indolepropionic acid, **(D)** Kynurenine, **(E)** Kynurenine to tryptophan, and **(F)** Kynurenine to tryptophan betaine in critical, severe, mild, and asymptomatic COVID-19 patients, and their association with patient outcomes. **(G)** ROC curve analysis of Kynurenine and Kynurenine to Tryptophan ratio, **(H)** Spearman correlation between kynurenine to tryptophan ratio and lymphocytes percentage, **(I)** Spearman correlation between kynurenine to tryptophan ratio and albumin, **(J)** COVID-19 survival probability in each high/low serotonin, **(K)** Kynurenine, **(L)** Tryptophan, **(M)** Tryptophan betaine, **(N)** Kynurenine to tryptophan ratio, **(O)** and Kynurenine to tryptophan betaine ratio. p > 0.05; *p ≤ 0.05; **p ≤ 0.01; ***p ≤ 0.001; ****p ≤ 0.0001.

Tryptophan is an essential amino acid that plays a vital role in protein synthesis, growth, mental health, and immune responses (40). Tryptophan pathway was among the top pathways that was impacted by SARS-CoV-2 severe infection. Our analysis revealed that tryptophan derivatives serotonin and tryptophan betaine were significantly reduced in the severe/critical group compared to mild and asymptomatic groups (**Figures 4A, B**) and this reduction was associated with disease severity. Previous study showed that the levels of tryptophan were significantly decreased in COVID-19 patients and were inversely correlated with IL-6 levels (41). It is well known that the essential amino acid tryptophan catabolism is tightly controlled by the rate-limiting enzyme indoleamine 2,3-dioxygenase (IDO) (37). IDO contributes to immune-metabolic regulation by depleting tryptophan or producing kynurenine, which both contributing to an increased susceptibility to infection (42). Our further analysis showed that 3-indolepropionic acid and kynurenine, the two tryptophan derived metabolites, and the ratios of kynurenine to tryptophan and kynurenine to tryptophan betaine were elevated (**Figures 4C, D**) in patients with severe/critical COVID-19 (**Figures 4E, F**). Additionally, receiver operating characteristic (ROC) curve analysis in our total cohort revealed an area under the curve (AUC) of 0.929 and 0.904 for kynurenine and kynurenine to tryptophan ratio respectively (**Figure 4G**). These results have shown good predictive value to discriminate between hospital deaths and survivors (**Figures 4C–G**). A significant negative correlation between kynurenine to tryptophan ratio and either lymphocyte percentage or albumin were observed with a Pearson coefficient of R=0.61 and 0.72 (p < 0.05), respectively (**Figures 4H, I**). In accordance with the alterations in metabolite levels mentioned above, patient survival probability in high serotonin (**Figure 4J**), tryptophan (**Figure 4L**), and tryptophan betaine (**Figure 4M**) groups was significantly improved compared to patients with low levels (p < 0.0001). Whereas high levels of kynurenine (**Figure 4K**) and elevated kynurenine:tryptophan (**Figure 4N**) and kynurenine:tryptophan betaine ratios (**Figure 4O**) were associated with decreased survival probability. Taken together, high levels of serotonin, tryptophan, and tryptophan betaine were associated with improved survival, while high levels of kynurenine, kynurenine to tryptophan ratio, and kynurenine to tryptophan betaine ratio were associated with decreased survival probability. Therefore, these metabolites warrant further testing as possible prognostic markers for severe/critical COVID-19 cases.

### 3.5 COVID-19 positive patients display alterations in carnitine metabolism

**Figure 5:**
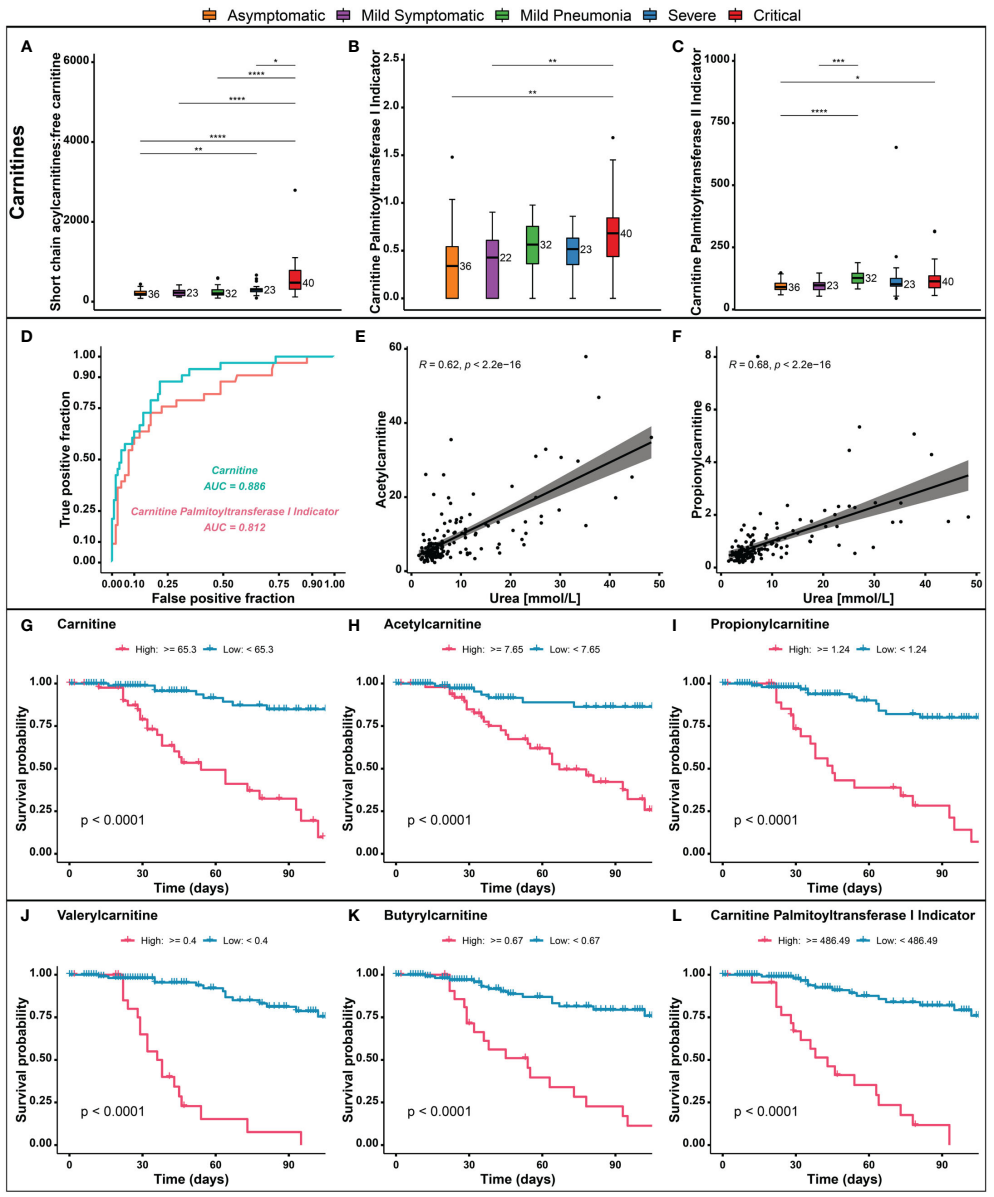
Metabolic markers in different COVID-19 severity groups. **(A)** Short chain acylcarnitines (SCACs). **(B, C)** Carnitine palmitoyltransferase 1 and 2 (CPT1 and CPT2) enzymes. **(D)** Receiver operating characteristic (ROC) curve analysis for carnitines and CPT1 indicators. **(E, F)** Correlation of acetylcarnitine and propionylcarnitine with urea. **(G–L)** Kaplan-Meier survival curves for carnitine, acylcarnitines, and CPT1. p > 0.05; *p ≤ 0.05; **p ≤ 0.01; ***p ≤ 0.001; ****p ≤ 0.0001.

The effects of SARS-CoV-2 infection on intermediary metabolism, including metabolism of acylcarnitines, has not been well studied. Nevertheless, measurement of total carnitine has been used as a precision biomarker to predict mortality risk in diseases such as sepsis, Type-2 diabetes, cancer, and heart failure (43). To better understand the dysregulation of acylcarnitine metabolism associated with the COVID-19 symptoms severity, we further analyzed our data for short-chain acylcarnitines (SCACs) concentration. Elevated levels of SCACs have been observed in critically ill COVID-19 patients, likely due to increased demand for energy, inflammation, and mitochondrial dysfunction (**Figure 5A**). Furthermore, elevated levels of carnitine palmitoyltransferase 1 and 2 (CPT1 and CPT2) enzymes, which are involved in fatty acid transport, have also been observed in critical COVID-19 patients as compared to asymptomatic (**Figures 5B, C**). ROC curve analysis demonstrated a high discriminatory power for carnitines and CPT1 indicator, with an area under the curve of 0.886 and 0.812 respectively, indicating their potential usefulness as predictors of hospital mortality in COVID-19 patients (**Figure 5D**). In addition, a positive correlation between acetylcarnitine and propionylcarnitine, the amino acid derivatives involved in fatty acid transport, and urea, a waste product of protein metabolism, was observed in COVID-19 patients (**Figures 5E, F**). Moreover, we utilized Kaplan-Meier survival curves to analyze the relationship between various metabolic markers and patient outcomes. Our analysis showed that patients with elevated levels of carnitine (**Figure 5G**), acylcarnitines (**Figures 5H–K**), and CPT1 (**Figure 5L**) markers had a poor survival outcome probability compared to those with normal levels. These findings suggest that these metabolic markers may have a potential prognostic value in COVID-19 patients and may be useful in the management of the disease.

### 3.6 Arginine metabolism and methylhistidines levels in non-survivors and severe cases

**Figure 6:**
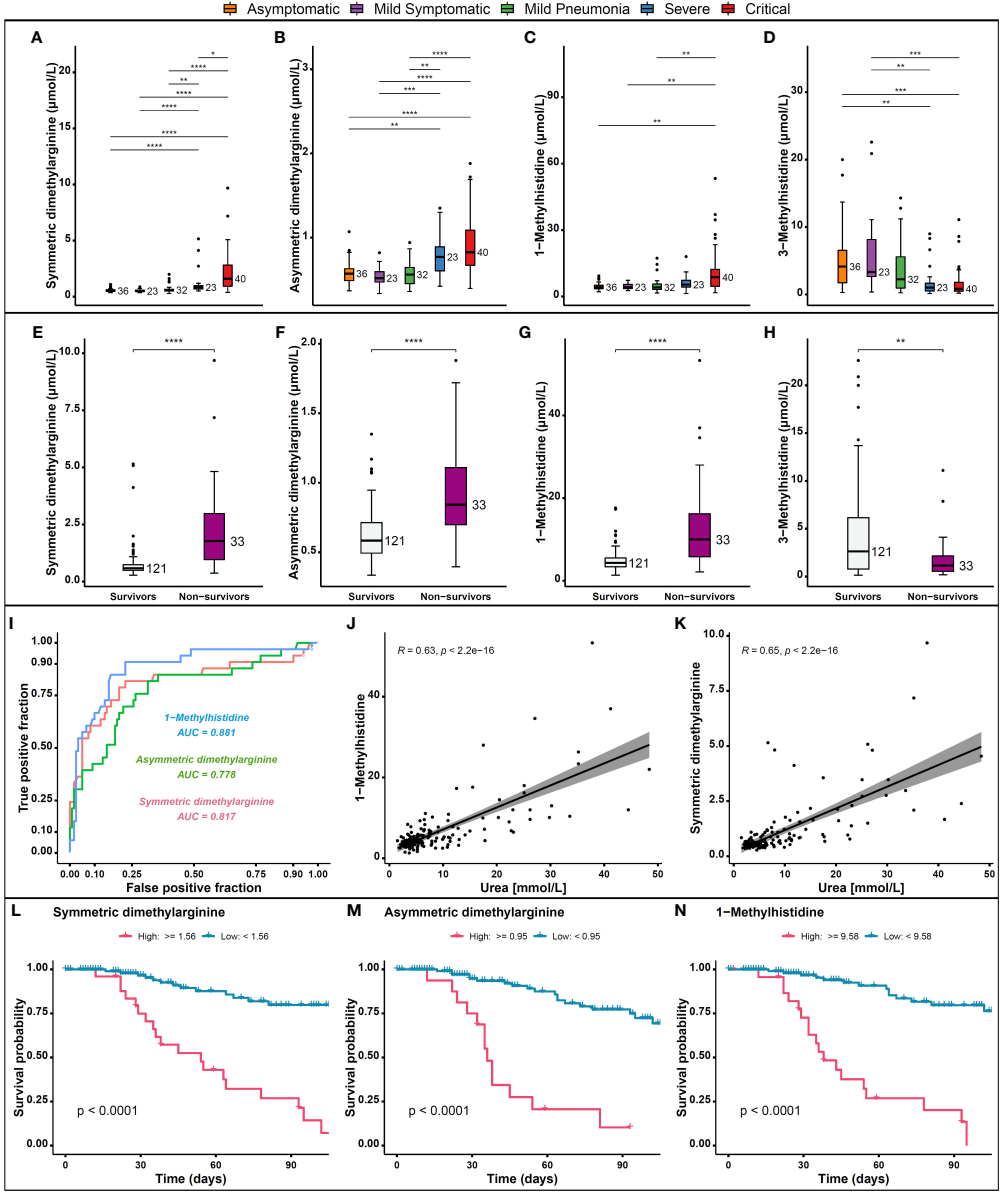
Levels of dimethylarginine and methylhistidine metabolites in COVID-19 patients and their association with outcomes. **(A)** Levels of SDMA, **(B)** ADMA, **(C)** 1-MH, and **(D)** 3-MH in patients with severe/critical disease compared to those with mild/moderate disease and asymptomatic cases. **(E)** Levels of SDMA, **(F)** ADMA, **(G)** 1-MH, and **(H)** 3-MH in non-survivors compared to survivors. **(I)** The AUC of SDMA, ADMA, 1-MH as biomarkers for patient outcomes in COVID-19. **(J)** Correlation between urea and 1-MH and **(K)** SDMA concentrations in COVID-19 patients. **(L)** Survival probabilities in relation to SDMA, **(M)** ADMA, and **(N)** 1-MH concentrations. p > 0.05; *p ≤ 0.05; **p ≤ 0.01; ***p ≤ 0.001; ****p ≤ 0.0001.

Recent reports show that arginine, one of the key amino acids involved in many different biological processes, could also play a crucial role in the COVID-19 infection (44). It is a substrate for nitric oxide (NO) synthase (NOS) (45) to generate NO, which is a major endothelial relaxation factor (46). In addition, arginine serves as a precursor for molecules such as SDMA and ADMA (46, 47). SDMA and ADMA are endogenous modulators of NO synthesis and intracellular arginine availability in the endothelium (48) and their circulating concentrations are known to be dysregulated in hypoxia (49). In addition, inhibition of NO synthesis by ADMA and SDMA may affect immune responses and inflammatory reaction, as they also interfere with inducible NO synthase, an enzyme that is upregulated by inflammatory cytokines (50). Furthermore, serum SDMA and ADMA were found to be significantly elevated in critical and severe COVID-19 patients, than in other groups and were significantly associated with disease severity (44). Our analyses showed that patients with severe/critical disease had significantly increased levels of SDMA and ADMA (**Figures 6A, B**) and 1-methylhistidine (1-MH) (**Figure 6C**), and non-survivors had even higher levels (**Figures 6E–G**). Conversely, 3-methylhistidine levels were lower in patients with severe disease (**Figure 6D**) and in non-survivors (**Figure 6H**). We also found that SDMA, ADMA, and 1-MH are good predictors of patient outcomes in COVID-19, as shown by the high discriminatory power with an AUC of 0.817, 0.778, and 0.881 respectively, which indicate their potential as useful biomarkers (**Figure 6I**). A positive correlation was observed between urea, 1-MH and SDMA levels in COVID-19 patients (**Figures 6J, K**). Consistent with other studies, survival probabilities were found to be higher when levels of SDMA, ADMA (**Figures 6L, M**) and 1-methylhistidine (**Figure 6N**) were reduced, confirming the association of ADMA, SDMA with COVID-19 mortality (51).

### 3.7 Machine learning models to predict mortality risk in Covid-19 patients

Machine learning (ML), a branch of artificial intelligence (AI) that learns from past data to build predictive models (52), has been applied in different fields in recent times, including medicine. ML is a useful tool to analyze large amounts of data from medical records including images (53) and to facilitate prediction of disease and clinical decision-making. Recent advances using ML in COVID-19 include estimation of mortality risk and prediction of progression to a severe or critical state and hospital stay duration (54). Although most studies have predicted the severity of COVID-19 disease and mortality risk using data from radiographic images (55–58), and laboratory findings (59), we have attempted to use metabolites data and ML to predict the severity of the disease and mortality risk in patients with COVID-19. We utilized a combination of four predictive biomarkers, namely tryptophan, kynurenine, asymmetric dimethylarginine, and 1-Methylhistidine to propose a mortality risk model. In this context, we used several machine learning methods including Random Forest, Support Vector Machines, Bernoulli Naıve Bayes, Gradient Boosting Classifiers, K-Nearest Neighbors, Neural Network, and Logistic Regression. The results showed that Random Forest yielded an accuracy of 96.77%, Support Vector Machine (SVM) 87.1%, Bernoulli Naive Bayes 87.1%, Gradient Boosting Classifier 100%, K-Nearest Neighbors 87.1%, Logistic Regression 93.55%, MLPClassifier 90.32% and Neural Network 90.32%. **Table 2** illustrates that the models were able to accurately predict the risk of mortality using a panel of four metabolomic markers with high sensitivity and specificity.

**Table 2:** Illustrates the results of accuracy of the ML models.

Model Name	Accuracy	True Positives	False Positives	False Negatives	True Negatives
Random Forest Classifier	96.77%	26	1	0	4
Support Vector Machine (SVM)	87.10%	26	1	3	1
Bernoulli Naive Bayes	87.10%	27	0	4	0
Gradient Boosting Classifier (XGBoost)	100%	27	0	0	4
K-Nearest Neighbors (KNN)	87.10%	24	3	1	3
Logistic Regression	93.55%	26	1	1	3
MLPClassifier (Multilayer Perceptron)	90.32%	25	2	1	3
Neural Network (Multilayer Perceptron)	90.32%	25	2	1	3

## 4 Discussion

Severe SARS-CoV-2 infection leads to changes in host metabolism promoting viral replication, and alterations in immune responses resulting in long‐term metabolic complications and sequelae in infected individuals (60). Emerging research provides compelling evidence that individuals experiencing severe SARS-CoV-2 infections often exhibit multiple metabolic disruptions. In this study, we employed Kaplan-Meier survival analysis to evaluate the correlation between metabolic markers that were previously reported and the patient outcomes. Our findings clearly revealed statistically significant differences in survival outcomes between individuals presenting with altered levels of several metabolites. To our knowledge our study is the first to identify four metabolites using multiple established machine learning models, which can help distinguish between COVID-19 clinical phenotypes and predict mortality risk.

A key finding reported by several COVID-19 studies is that many amino acids and their related metabolites are dysregulated following severe COVID-19 infection, the majority of which are significantly downregulated (61, 62). Compared to these studies, in our cohort, significant differences in serum amino acid levels between different severity groups and survivors and non-survivors were observed. Amino acids play a key role in immune cell function, tissue regeneration and repair, while an abnormal amino acid metabolism could cause neurological symptoms and multi-organ failure (63). It is reported that recovered COVID-19 patients have a certain degree of neurological sequelae and patients with severe COVID-19, may develop multi-organ failure during hospitalization (25, 27). We found that several amino acids and their associated metabolites such as alanine, tryptophan, serine, glutamine, and histidine were significantly reduced, while phenylalanine and tyrosine were upregulated in severe and critical COVID-19 cases. These amino acids are key players in energy metabolism, neurotransmitter production and metabolic homeostasis regulation (64, 65). Multiple studies have shown that COVID-19 patients had an enriched levels of taurine and hypotaurine metabolic pathways (27, 66, 67) indicating that an overactive taurine pathway may drive the excessive immune responses. Therefore, amino acid pathways could be promising targets for drug development.

Other amino acids, including tryptophan derivatives, serotonin and tryptophan betaine, 3-indolepropionic acid, and kynurenine remain dysregulated in the severe/critical group. Multiple studies have revealed that the metabolome of COVID-19 patients, including products of the tryptophan/kynurenine pathway, reflects the severity of the disease and thus can be used to predict disease evolution (12, 68, 69). It has been shown that Interleukin-6 (IL-6) levels were linked to tryptophan metabolism (26). Furthermore, kynurenine and arginine are known to be essential for the immunosuppressive activity of dendritic cells, which are critical immunomodulators (70). Consistent with other studies, severe and critical COVID-19 patients showed increased levels of oxidative stress markers, dysregulation of tryptophan metabolism, and renal dysfunction, which correlated with the decreased lymphocyte count (71, 72). Indeed, several metabolite levels in tryptophan pathway correlated with clinical laboratory markers of inflammation and renal function (26). Thus, their persistent dysregulation is most likely linked to the underlying molecular mechanism of long-term COVID-19 and requires further investigation and targeted interventions.

It is also worth noting that significantly elevated levels of short chain acylcarnitines and CPT1 were observed in accord with disease progression. Carnitine is a vitamin-like compound that plays an important role in fatty acid metabolism (73). It is mainly synthesized in the brain, liver and kidney and is primarily stored in the skeletal muscle and heart (74). Elevated acylcarnitines in COVID-19 patients have been proposed as activators of pro-inflammatory pathways (75), and their imbalance has been related to ATP depletion (76). Our results support the idea that COVID-19 patients present an over utilization of lipid beta-oxidation pathway to supply to the high energetic demand (66). Thus, this could also suggest dysregulation of these metabolites especially, the short chain acylcarnitines, which are fundamental for maintaining optimal energy metabolism. Furthermore, random forest analysis revealed that carnitine, acylcarnitines and CPT1 show excellent performance in survival outcome probability for COVID-19 patients. This suggests that understanding the metabolic changes of carnitine, acylcarnitines and CPT1 during COVID-19 may advance monitoring disease progression and have a potential prognostic value.

An increase in ferritin level was observed and non-survivors had higher serum ferritin level compared to survivors, confirming enrichment of ferroptosis and energy metabolism pathways in patients with COVID-19. The serum of patients with COVID-19 showed an iron imbalance (77) and significantly elevated ferritin levels were related to disease severity, development of acute respiratory distress syndrome (ARDS) and death in COVID-19 patients (78–81). Indeed, serum ferritin has recently been identified as one of the predictors of death in COVID-19 patients (82–84). Furthermore, a recent study has demonstrated that COVID-19 infection causes hemoglobin damage (85). Consequently, this leads to detachment of porphyrins from iron, release of iron into the circulation resulting in iron overload and subsequent elevation of ferritin levels (80). Ferritin is a key mediator of immune dysregulation, especially under extreme hyperferritinemia, via direct immune-suppressive and pro-inflammatory effects, which contribute to cytokine storm and multi-organ damage and failure (86, 87).

ML has been demonstrated to play a significant role in understanding and combating the pandemic, particularly in predicting mortality risk and severity based on laboratory test results. In the present study, we employed multiple well-established ML models to predict the mortality risk model for COVID-19 patients, based on altered metabolites identified through our data and recent literature. This highlights the potential for ML models to provide a clinically valuable tool in predicting mortality risk in COVID-19 patients based on their metabolomic profile and suggests that research efforts should not overlook metabolic signatures of the disease.

A significant contribution in our study is the incorporation of five distinct severity categories, a characteristic less prevalent or non-present in the majority of existing published research. This distinctive approach allows us to explore a broader spectrum of disease progression, enhancing the granularity of our analysis. Additionally, our study uniquely identifies elevated serum SDMA and ADMA concentrations within these severity groups, rather than merely distinguishing between deceased and surviving patients. This study also presents innovative dimensions, including the integration of Kaplan-Meier survival curves and machine learning techniques to evaluate patient outcomes and predict mortality risk. We emphasize the significance of the Kaplan-Meier survival curves in our research, as they provide a dynamic perspective on the correlation between a diverse array of metabolic markers and COVID-19 patient outcomes across time.

The limitation of this study is that the samples used were collected during the early days of the pandemic, which may not reflect the status of vaccinated population cohort that is prevalent. This could potentially impact the generalization of the findings to more recent cases, as the vaccination may impact the metabolic changes observed. We also acknowledge that practical constraints, such as limited sample availability, had led to unequal distributions of samples among different groups. As a limitation, it should be noted that the small number of female participants in our study (n=16) may limit the statistical power to draw definitive conclusions about potential differences between sexes. Future studies with a larger and more balanced representation of both genders can provide more robust insights into gender-specific variations in COVID-19 disease outcomes. Further research is required to better understand the underlying mechanisms of the relationship between these metabolic markers and COVID-19 outcomes.

As a conclusion, the metabolomic fingerprint of COVID-19 related to disease progression is characterized by dysregulation of amino acids and short chain acylcarnitines metabolic pathways, particularly tryptophan and arginine, and fatty acid metabolism. Our data suggest that metabolic dysregulation could induce states of hypoxemia, ferroptosis and other clinical characteristics of COVID-19. The dysregulation of amino acids and metabolites including tryptophan, kynurenine, carnitine, arginine SDMA and ADMA observed in our study was clearly associated with critical outcomes in COVID-19 patients. Therefore, these metabolites could be considered as promising biomarkers to identify patients at risk of poor outcomes in COVID-19. In addition, short chain acylcarnitines and carnitine palmitoyltransferase 1 and 2 enzymes indicators could be considered as promising predictors of hospital mortality and poor survival outcome. Importantly, high serum ferritin level was found to be associated with more severe disease and negative/poor outcome in COVID-19. Thus, serum ferritin level can serve as an important predictive biomarker in COVID-19 management.

Altogether, these findings hold the potential to serve as prognostic markers, aiding in the assessment of disease severity and the prediction of patient outcomes. Notably, the identification of specific metabolites linked to disease progression and mortality risk contribute to more informed clinical decision-making, ultimately enhancing patient care and management strategies as well as COVID-19 prognosis and treatment.

## Data availability statement

The original contributions presented in the study are included in the article/**Supplementary Material**. Further inquiries can be directed to the corresponding authors.

## Ethics statement

The studies involving humans were approved by the studies involving human participants were reviewed and approved by IRBs at Hamad Medical Corporation and Qatar University. The patients/participants provided their written informed consent to participate in this study. The studies were conducted in accordance with the local legislation and institutional requirements. Written informed consent for participation in this study was provided by the participants’ legal guardians/next of kin.

## Author contributions

AA: Conceptualization, Funding acquisition, Project administration, Supervision, Writing – original draft, Writing – review & editing, Resources. AD: Formal analysis, Methodology, Writing – review & editing. TS: Methodology, Writing – review & editing. OJ: Methodology, Writing – review & editing. RA: Methodology, Writing – review & editing. FA: Investigation, Writing – review & editing. HY: Funding acquisition, Writing – review & editing. MAE: Methodology, Writing – review & editing. A-NE: Investigation, Writing – review & editing. MME: Investigation, Writing – review & editing. NT: Investigation, Visualization, Writing – review & editing. FC: Funding acquisition, Conceptualization, Investigation, Methodology, Supervision, Validation, Visualization, Writing – original draft, Writing – review & editing.

## References

[1] Ali I Alharbi OML. COVID-19: Disease, management, treatment, and social impact. Sci Total Environ. (2020) 728:138861. doi: 10.1016/j.scitotenv.2020.138861 32344226 PMC7175909

[2] Phelan AL Katz R Gostin LO. The novel coronavirus originating in wuhan, China: challenges for global health governance. JAMA. (2020) 323:709–10. doi: 10.1001/jama.2020.1097 31999307

[3] WHO. Weekly epidemiological update on COVID-19 - 25 May 2023 (2023). Available online at: https://www.who.int/publications/m/item/weekly-epidemiological-update-on-covid-19—25-may-2023.

[4] Fu L Wang B Yuan T Chen X Ao Y Fitzpatrick T. Clinical characteristics of coronavirus disease 2019 (COVID-19) in China: A systematic review and meta-analysis. J Infect. (2020) 80:656–65. doi: 10.1016/j.jinf.2020.03.041 PMC7151416 32283155

[5] Nishiura H Kobayashi T Miyama T Suzuki A Jung SM Hayashi K. Estimation of the asymptomatic ratio of novel coronavirus infections (COVID-19). Int J Infect Dis. (2020) 94:154–5. doi: 10.1016/j.ijid.2020.03.020 PMC7270890 32179137

[6] Williamson EJ Walker AJ Bhaskaran K Bacon S Bates C Morton CE. Factors associated with COVID-19-related death using OpenSAFELY. Nature. (2020) 584:430–6. doi: 10.1038/s41586-020-2521-4 PMC7611074 32640463

[7] Cao H Baranova A Wei X Wang C Zhang F. Bidirectional causal associations between type 2 diabetes and COVID-19. J Med Virol. (2023) 95:e28100. doi: 10.1002/jmv.28100 36029131 PMC9538258

[8] Baranova A Cao H Teng S Zhang F. A phenome-wide investigation of risk factors for severe COVID-19. J Med Virol. (2023) 95:e28264. doi: 10.1002/jmv.28264 36316288 PMC9874597

[9] Baranova A Cao H Zhang F. Causal associations and shared genetics between hypertension and COVID-19. J Med Virol. (2023) 95:e28698. doi: 10.1002/jmv.28698 36951353

[10] Polack FP Thomas SJ Kitchin N Absalon J Gurtman A Lockhart S. Safety and efficacy of the BNT162b2 mRNA covid-19 vaccine. N Engl J Med. (2020) 383:2603–15. doi: 10.1056/NEJMoa2034577 PMC7745181 33301246

[11] Voysey M Clemens SAC Madhi SA Weckx LY Folegatti PM Aley PK. Safety and efficacy of the ChAdOx1 nCoV-19 vaccine (AZD1222) against SARS-CoV-2: an interim analysis of four randomised controlled trials in Brazil, South Africa, and the UK. Lancet. (2021) 397:99–111. doi: 10.1016/S0140-6736(20)32661-1 33306989 PMC7723445

[12] Blasco H Bessy C Plantier L Lefevre A Piver E Bernard L. The specific metabolome profiling of patients infected by SARS-COV-2 supports the key role of tryptophan-nicotinamide pathway and cytosine metabolism. Sci Rep. (2020) 10:16824. doi: 10.1038/s41598-020-73966-5 33033346 PMC7544910

[13] Denson JL Gillet AS Zu Y Brown M Pham T Yoshida Y. Metabolic syndrome and acute respiratory distress syndrome in hospitalized patients with COVID-19. JAMA Netw Open. (2021) 4:e2140568. doi: 10.1001/jamanetworkopen.2021.40568 34935924 PMC8696573

[14] Holmes E Wilson ID Nicholson JK. Metabolic phenotyping in health and disease. Cell. (2008) 134:714–7. doi: 10.1016/j.cell.2008.08.026 18775301

[15] Skene DJ Middleton B Fraser CK Pennings JL Kuchel TR Rudiger SR. Metabolic profiling of presymptomatic Huntington’s disease sheep reveals novel biomarkers. Sci Rep. (2017) 7:43030. doi: 10.1038/srep43030 28223686 PMC5320451

[16] Caterino M Gelzo M Sol S Fedele R Annunziata A Calabrese C. Dysregulation of lipid metabolism and pathological inflammation in patients with COVID-19. Sci Rep. (2021) 11:2941. doi: 10.1038/s41598-021-82426-7 33536486 PMC7859398

[17] Collier ME Zhang S Scrutton NS Giorgini F. Inflammation control and improvement of cognitive function in COVID-19 infections: is there a role for kynurenine 3-monooxygenase inhibition? Drug Discovery Today. (2021) 26:1473–81. doi: 10.1016/j.drudis.2021.02.009 PMC7889466 33609782

[18] Danlos FX Grajeda-Iglesias C Durand S Sauvat A Roumier M Cantin D. Metabolomic analyses of COVID-19 patients unravel stage-dependent and prognostic biomarkers. Cell Death Dis. (2021) 12:258. doi: 10.1038/s41419-021-03540-y 33707411 PMC7948172

[19] Lawler NG Gray N Kimhofer T Boughton B Gay M Yang R. Systemic perturbations in amine and kynurenine metabolism associated with acute SARS-coV-2 infection and inflammatory cytokine responses. J Proteome Res. (2021) 20:2796–811. doi: 10.1021/acs.jproteome.1c00052 33724837

[20] Lopez-Hernandez Y Monarrez-Espino J Oostdam AH Delgado JEC Zhang L Zheng J. Targeted metabolomics identifies high performing diagnostic and prognostic biomarkers for COVID-19. Sci Rep. (2021) 11:14732. doi: 10.1038/s41598-021-94171-y 34282210 PMC8290000

[21] Overmyer KA Shishkova E Miller IJ Balnis J Bernstein MN Peters-Clarke TM. Large-scale multi-omic analysis of COVID-19 severity. Cell Syst. (2021) 12:23–40 e27. doi: 10.1016/j.cels.2020.10.003 33096026 PMC7543711

[22] Pang Z Zhou G Chong J Xia J. Comprehensive meta-analysis of COVID-19 global metabolomics datasets. Metabolites. (2021) 11(1):44. doi: 10.3390/metabo11010044 33435351 PMC7827862

[23] Schroeder M Schaumburg B Mueller Z Parplys A Jarczak D Roedl K. High estradiol and low testosterone levels are associated with critical illness in male but not in female COVID-19 patients: a retrospective cohort study. Emerg Microbes Infect. (2021) 10:1807–18. doi: 10.1080/22221751.2021.1969869 PMC8451658 34402750

[24] Xiao N Nie M Pang H Wang B Hu J Meng X. Integrated cytokine and metabolite analysis reveals immunometabolic reprogramming in COVID-19 patients with therapeutic implications. Nat Commun. (2021) 12:1618. doi: 10.1038/s41467-021-21907-9 33712622 PMC7955129

[25] Kimhofer T Lodge S Whiley L Gray N Loo RL Lawler NG. Integrative modeling of quantitative plasma lipoprotein, metabolic, and amino acid data reveals a multiorgan pathological signature of SARS-coV-2 infection. J Proteome Res. (2020) 19:4442–54. doi: 10.1021/acs.jproteome.0c00519 32806897

[26] Thomas T Stefanoni D Reisz JA Nemkov T Bertolone L Francis RO. COVID-19 infection alters kynurenine and fatty acid metabolism, correlating with IL-6 levels and renal status. JCI Insight. (2020) 5(14):e140327. doi: 10.1172/jci.insight.140327 32559180 PMC7453907

[27] Rees CA Rostad CA Mantus G Anderson EJ Chahroudi A Jaggi P. Altered amino acid profile in patients with SARS-CoV-2 infection. Proc Natl Acad Sci U.S.A. (2021) 118(25):e2101708118. doi: 10.1073/pnas.2101708118 34088793 PMC8237604

[28] Bharadwaj S Singh M Kirtipal N Kang SG. SARS-CoV-2 and glutamine: SARS-CoV-2 triggered pathogenesis via metabolic reprograming of glutamine in host cells. Front Mol Biosci. (2020) 7:627842. doi: 10.3389/fmolb.2020.627842 33585567 PMC7873863

[29] Matsuyama T Yoshinaga SK Shibue K Mak TW. Comorbidity-associated glutamine deficiency is a predisposition to severe COVID-19. Cell Death Differ. (2021) 28:3199–213. doi: 10.1038/s41418-021-00892-y PMC8522258 34663907

[30] Wu D Shu T Yang X Song JX Zhang M Yao C. Plasma metabolomic and lipidomic alterations associated with COVID-19. Natl Sci Rev. (2020) 7:1157–68. doi: 10.1093/nsr/nwaa086 PMC7197563 34676128

[31] WHO. COVID-19: symptoms (2023). Available online at: https://www.who.int/westernpacific/emergencies/covid-19/information/asymptomatic-covid-19#:~:text=Symptoms%20of%20COVID%2D19%20can,should%20seek%20immediate%20medical%20attention.

[32] Elrayess MA Cyprian FS Abdallah AM Emara MM Diboun I Anwardeen N. Metabolic signatures of type 2 diabetes mellitus and hypertension in COVID-19 patients with different disease severity. Front Med (Lausanne). (2021) 8:788687. doi: 10.3389/fmed.2021.788687 35083246 PMC8784560

[33] Diboun I Cyprian FS Anwardeen NR Yassine HM Elrayess MA Rahmoon SM. Identification of prognostic metabolomic biomarkers at the interface of mortality and morbidity in pre-existing TB cases infected with SARS-coV-2. Front Cell Infect Microbiol. (2022) 12:929689. doi: 10.3389/fcimb.2022.929689 35937683 PMC9354137

[34] Herold T Jurinovic V Arnreich C Lipworth BJ Hellmuth JC von Bergwelt-Baildon M. Elevated levels of IL-6 and CRP predict the need for mechanical ventilation in COVID-19. J Allergy Clin Immunol. (2020) 146:128–136 e124. doi: 10.1016/j.jaci.2020.05.008 32425269 PMC7233239

[35] Morrison T Watts ER Sadiku P Walmsley SR. The emerging role for metabolism in fueling neutrophilic inflammation. Immunol Rev. (2023) 314:427–41. doi: 10.1111/imr.13157 PMC10953397 36326284

[36] Li Y Hook JS Ding Q Xiao X Chung SS Mettlen M. Neutrophil metabolomics in severe COVID-19 reveal GAPDH as a suppressor of neutrophil extracellular trap formation. Nat Commun. (2023) 14:2610. doi: 10.1038/s41467-023-37567-w 37147288 PMC10162006

[37] Munn DH Shafizadeh E Attwood JT Bondarev I Pashine A Mellor AL. Inhibition of T cell proliferation by macrophage tryptophan catabolism. J Exp Med. (1999) 189:1363–72. doi: 10.1084/jem.189.9.1363 PMC2193062 10224276

[38] Maltais-Payette I Lajeunesse-Trempe F Pibarot P Biertho L Tchernof A. Association between circulating amino acids and COVID-19 severity. Metabolites. (2023) 13(2):201. doi: 10.3390/metabo13020201 36837819 PMC9959167

[39] Mason S van Reenen M Rossouw T Lindeque Z Louw R. Phenylalanine metabolism and tetrahydrobiopterin bio-availability in COVID-19 and HIV. Heliyon. (2023) 9:e15010. doi: 10.1016/j.heliyon.2023.e15010 37009248 PMC10043972

[40] Xue C Li G Zheng Q Gu X Shi Q Su Y. Tryptophan metabolism in health and disease. Cell Metab. (2023) 35(8):1304–26. doi: 10.1016/j.cmet.2023.06.004 37352864

[41] Masoodi M Peschka M Schmiedel S Haddad M Frye M Maas C. Disturbed lipid and amino acid metabolisms in COVID-19 patients. J Mol Med (Berl). (2022) 100:555–68. doi: 10.1007/s00109-022-02177-4 PMC8783191 35064792

[42] Munn DH Mellor AL. Indoleamine 2,3 dioxygenase and metabolic control of immune responses. Trends Immunol. (2013) 34:137–43. doi: 10.1016/j.it.2012.10.001 PMC3594632 23103127

[43] McCann MR George de la Rosa MV Rosania GR Stringer KA. L-carnitine and acylcarnitines: mitochondrial biomarkers for precision medicine. Metabolites. (2021) 11(1):51. doi: 10.3390/metabo11010051 33466750 PMC7829830

[44] Sozio E Hannemann J Fabris M Cifu A Ripoli A Sbrana F. The role of asymmetric dimethylarginine (ADMA) in COVID-19: association with respiratory failure and predictive role for outcome. Sci Rep. (2023) 13:9811. doi: 10.1038/s41598-023-36954-z 37330534 PMC10276836

[45] Andrew PJ Mayer B. Enzymatic function of nitric oxide synthases. Cardiovasc Res. (1999) 43:521–31. doi: 10.1016/s0008-6363(99)00115-7 10690324

[46] Gambardella J Khondkar W Morelli MB Wang X Santulli G Trimarco V. Arginine and endothelial function. Biomedicines. (2020) 8(8):277. doi: 10.3390/biomedicines8080277 32781796 PMC7460461

[47] Hsu CN Tain YL. Impact of arginine nutrition and metabolism during pregnancy on offspring outcomes. Nutrients. (2019) 11(7):1452. doi: 10.3390/nu11071452 31252534 PMC6682918

[48] Boger RH. The emerging role of asymmetric dimethylarginine as a novel cardiovascular risk factor. Cardiovasc Res. (2003) 59:824–33. doi: 10.1016/s0008-6363(03)00500-5 14553822

[49] Hannemann J Zummack J Hillig J Boger R. Metabolism of asymmetric dimethylarginine in hypoxia: from bench to bedside. Pulm Circ. (2020) 10:2045894020918846. doi: 10.1177/2045894020918846 32313644 PMC7158260

[50] Boger RH. Live and let die: asymmetric dimethylarginine and septic shock. Crit Care. (2006) 10:169. doi: 10.1186/cc5076 17094795 PMC1794448

[51] Hannemann J Balfanz P Schwedhelm E Hartmann B Ule J Muller-Wieland D. Elevated serum SDMA and ADMA at hospital admission predict in-hospital mortality of COVID-19 patients. Sci Rep. (2021) 11:9895. doi: 10.1038/s41598-021-89180-w 33972591 PMC8110746

[52] Van Calster B Wynants L. Machine learning in medicine. N Engl J Med. (2019) 380:2588. doi: 10.1056/NEJMc1906060 31242379

[53] Lambin P Leijenaar RTH Deist TM Peerlings J de Jong EEC van Timmeren J. Radiomics: the bridge between medical imaging and personalized medicine. Nat Rev Clin Oncol. (2017) 14:749–62. doi: 10.1038/nrclinonc.2017.141 28975929

[54] Yue H Yu Q Liu C Huang Y Jiang Z Shao C. Machine learning-based CT radiomics method for predicting hospital stay in patients with pneumonia associated with SARS-CoV-2 infection: a multicenter study. Ann Transl Med. (2020) 8:859. doi: 10.21037/atm-20-3026 32793703 PMC7396749

[55] Alakus TB Turkoglu I. Comparison of deep learning approaches to predict COVID-19 infection. Chaos Solitons Fractals. (2020) 140:110120. doi: 10.1016/j.chaos.2020.110120 33519109 PMC7833512

[56] Cohen JP Dao L Roth K Morrison P Bengio Y Abbasi AF. Predicting COVID-19 pneumonia severity on chest X-ray with deep learning. Cureus. (2020) 12:9448. doi: 10.7759/cureus.9448 PMC7451075 32864270

[57] Liang W Yao J Chen A Lv Q Zanin M Liu J. Early triage of critically ill COVID-19 patients using deep learning. Nat Commun. (2020) 11:3543. doi: 10.1038/s41467-020-17280-8 32669540 PMC7363899

[58] Fernandez A ObieChina N Koh J Hong A Nandi A Reynolds TM. Survival prediction algorithms for COVID-19 patients admitted to a UK district general hospital. Int J Clin Pract. (2021) 75:e13974. doi: 10.1111/ijcp.13974 33368796 PMC7883072

[59] Taheriyan M Ayyoubzadeh SM Ebrahimi M Niakan Kalhori SR Abooei AH Gholamzadeh M. Prediction of COVID-19 patients’ Survival by deep learning approaches. Med J Islam Repub Iran. (2022) 36:144. doi: 10.47176/mjiri.36.144 36569399 PMC9774992

[60] Ayres JS. A metabolic handbook for the COVID-19 pandemic. Nat Metab. (2020) 2:572–85. doi: 10.1038/s42255-020-0237-2 PMC7325641 32694793

[61] Shen B Yi X Sun Y Bi X Du J Zhang C. Proteomic and metabolomic characterization of COVID-19 patient sera. Cell. (2020) 182:59–72 e15. doi: 10.1016/j.cell.2020.05.032 32492406 PMC7254001

[62] Atila A Alay H Yaman ME Akman TC Cadirci E Bayrak B. The serum amino acid profile in COVID-19. Amino Acids. (2021) 53:1569–88. doi: 10.1007/s00726-021-03081-w PMC8487804 34605988

[63] Wu G. Amino acids: metabolism, functions, and nutrition. Amino Acids. (2009) 37:1–17. doi: 10.1007/s00726-009-0269-0 19301095

[64] Petersen KF Dufour S Cline GW Shulman GI. Regulation of hepatic mitochondrial oxidation by glucose-alanine cycling during starvation in humans. J Clin Invest. (2019) 129:4671–5. doi: 10.1172/JCI129913 PMC6819088 31545298

[65] Waskiw-Ford M Hannaian S Duncan J Kato H Abou Sawan S Locke M. Leucine-enriched essential amino acids improve recovery from post-exercise muscle damage independent of increases in integrated myofibrillar protein synthesis in young men. Nutrients. (2020) 12(4):1061. doi: 10.3390/nu12041061 32290521 PMC7231404

[66] Paez-Franco JC Torres-Ruiz J Sosa-Hernandez VA Cervantes-Diaz R Romero-Ramirez S Perez-Fragoso A. Metabolomics analysis reveals a modified amino acid metabolism that correlates with altered oxygen homeostasis in COVID-19 patients. Sci Rep. (2021) 11:6350. doi: 10.1038/s41598-021-85788-0 33737694 PMC7973513

[67] Philips AM Khan N. Amino acid sensing pathway: A major check point in the pathogenesis of obesity and COVID-19. Obes Rev. (2021) 22:e13221. doi: 10.1111/obr.13221 33569904 PMC7995014

[68] Cai Y Kim DJ Takahashi T Broadhurst DI Yan H Ma S. Kynurenic acid may underlie sex-specific immune responses to COVID-19. Sci Signal. (2021) 14(690):eabf8483. doi: 10.1126/scisignal.abf8483 34230210 PMC8432948

[69] Federica G Giuseppina F Veronica L Gianpaolo Z Massimo T Veronica M. An untargeted metabolomic approach to investigate antiviral defence mechanisms in memory leukocytes secreting anti-SARS-CoV-2 IgG *in vitro* . Sci Rep. (2023) 13:629. doi: 10.1038/s41598-022-26156-4 36635345 PMC9835734

[70] Mondanelli G Bianchi R Pallotta MT Orabona C Albini E Iacono A. A relay pathway between arginine and tryptophan metabolism confers immunosuppressive properties on dendritic cells. Immunity. (2017) 46:233–44. doi: 10.1016/j.immuni.2017.01.005 PMC5337620 28214225

[71] Dewulf JP Martin M Marie S Oguz F Belkhir L De Greef J. Urine metabolomics links dysregulation of the tryptophan-kynurenine pathway to inflammation and severity of COVID-19. Sci Rep. (2022) 12:9959. doi: 10.1038/s41598-022-14292-w 35705608 PMC9198612

[72] Valdes A Moreno LO Rello SR Orduna A Bernardo D Cifuentes A. Metabolomics study of COVID-19 patients in four different clinical stages. Sci Rep. (2022) 12:1650. doi: 10.1038/s41598-022-05667-0 35102215 PMC8803913

[73] Hanai T Shiraki M Imai K Suetugu A Takai K Shimizu M. Usefulness of carnitine supplementation for the complications of liver cirrhosis. Nutrients. (2020) 12(7):1. doi: 10.3390/nu12071915 PMC7401279 32610446

[74] Vaz FM Wanders RJ. Carnitine biosynthesis in mammals. Biochem J. (2002) 361:417–29. doi: 10.1042/0264-6021:3610417 PMC1222323 11802770

[75] Herrera-Van Oostdam AS Castaneda-Delgado JE Oropeza-Valdez JJ Borrego JC Monarrez-Espino J Zheng J. Immunometabolic signatures predict risk of progression to sepsis in COVID-19. PloS One. (2021) 16:e0256784. doi: 10.1371/journal.pone.0256784 34460840 PMC8405033

[76] Thomas T Stefanoni D Dzieciatkowska M Issaian A Nemkov T Hill RC. Evidence of structural protein damage and membrane lipid remodeling in red blood cells from COVID-19 patients. J Proteome Res. (2020) 19:4455–69. doi: 10.1021/acs.jproteome.0c00606 PMC7640979 33103907

[77] Zhao K Huang J Dai D Feng Y Liu L Nie S. Serum iron level as a potential predictor of coronavirus disease 2019 severity and mortality: A retrospective study. Open Forum Infect Dis. (2020) 7:ofaa250. doi: 10.1093/ofid/ofaa250 32661499 PMC7337740

[78] Henry BM de Oliveira MHS Benoit S Plebani M Lippi G. Hematologic, biochemical and immune biomarker abnormalities associated with severe illness and mortality in coronavirus disease 2019 (COVID-19): a meta-analysis. Clin Chem Lab Med. (2020) 58:1021–8. doi: 10.1515/cclm-2020-0369 32286245

[79] Phua J Weng L Ling L Egi M Lim CM Divatia JV. Intensive care management of coronavirus disease 2019 (COVID-19): challenges and recommendations. Lancet Respir Med. (2020) 8:506–17. doi: 10.1016/S2213-2600(20)30161-2 PMC7198848 32272080

[80] Zhou F Yu T Du R Fan G Liu Y Liu Z. Clinical course and risk factors for mortality of adult inpatients with COVID-19 in Wuhan, China: a retrospective cohort study. Lancet. (2020) 395:1054–62. doi: 10.1016/S0140-6736(20)30566-3 PMC7270627 32171076

[81] Wu C Chen X Cai Y Xia J Zhou X Xu S. Risk factors associated with acute respiratory distress syndrome and death in patients with coronavirus disease 2019 pneumonia in wuhan, China. JAMA Intern Med. (2020) 180:934–43. doi: 10.1001/jamainternmed.2020.0994 PMC7070509 32167524

[82] Kernan KF Carcillo JA. Hyperferritinemia and inflammation. Int Immunol. (2017) 29:401–9. doi: 10.1093/intimm/dxx031 PMC5890889 28541437

[83] Hadi JM Mohammad HM Ahmed AY Tofiq SS Abdalrahman LB Qasm AA. Investigation of serum ferritin for the prediction of COVID-19 severity and mortality: A cross-sectional study. Cureus. (2022) 14:e31982. doi: 10.7759/cureus.31982 36589200 PMC9797152

[84] Kaushal K Kaur H Sarma P Bhattacharyya A Sharma DJ Prajapat M. Serum ferritin as a predictive biomarker in COVID-19. A systematic review, meta-analysis and meta-regression analysis. J Crit Care. (2022) 67:172–81. doi: 10.1016/j.jcrc.2021.09.023 PMC8604557 34808527

[85] Habib HM Ibrahim S Zaim A Ibrahim WH. The role of iron in the pathogenesis of COVID-19 and possible treatment with lactoferrin and other iron chelators. BioMed Pharmacother. (2021) 136:111228. doi: 10.1016/j.biopha.2021.111228 33454595 PMC7836924

[86] Vargas-Vargas M Cortes-Rojo C. Ferritin levels and COVID-19. Rev Panam Salud Publica. (2020) 44:e72. doi: 10.26633/RPSP.2020.72 32547616 PMC7286435

[87] Fratta Pasini AM Stranieri C Girelli D Busti F Cominacini L. Is ferroptosis a key component of the process leading to multiorgan damage in COVID-19? Antioxid (Basel). (2021) 10(11):1677. doi: 10.3390/antiox10111677 PMC8615234 34829548

