# Supplementary material for: Metabolic predictors of COVID-19 mortality and severity: a survival analysis
Source: Front Immunol. 2024 May 10;15:1353903. doi: 10.3389/fimmu.2024.1353903 (PMC11127595; doi:10.3389/fimmu.2024.1353903)
Supplement: Supplementary file 1 [file DataSheet_1.pdf]

Supplementary Material

Metabolic predictors of COVID-19 mortality and severity: A Survival Analysis

Abdallah Musa Abdallah[1*], Asmma Doudin[2], Theeb Sulaiman[3], Omar Jamil[4], Rida Arif[5], Fatima Al Saada[2], Hadi Yassine[2], Mohamed A. Elrayess[1,2], Abdel-Naser Elzouki[1,3], Mohamed M. Emara[1], Nagendra Babu Thillaiappan[1], Farhan S. Cyprian[1*]

[1]College of Medicine, QU Health, Qatar University, Doha, Qatar; [2]Biomedical Research Center (BRC), Qatar University, Doha, Qatar; [3]Internal Medicine Department, Hamad General Hospital, Hamad Medical Corporation, Doha, Qatar; [4]Department of Radiology, Hamad General Hospital, Hamad Medical Corporation, Doha, Qatar; [5]Emergency Medicine Department, Hamad General Hospital, Hamad Medical Corporation, Doha, Qatar.

Correspondence*: Abdallah Musa Abdallah & Farhan S. Cyprian

Abdallah.musa@qu.edu.qa

fcyprian@qu.edu.qa

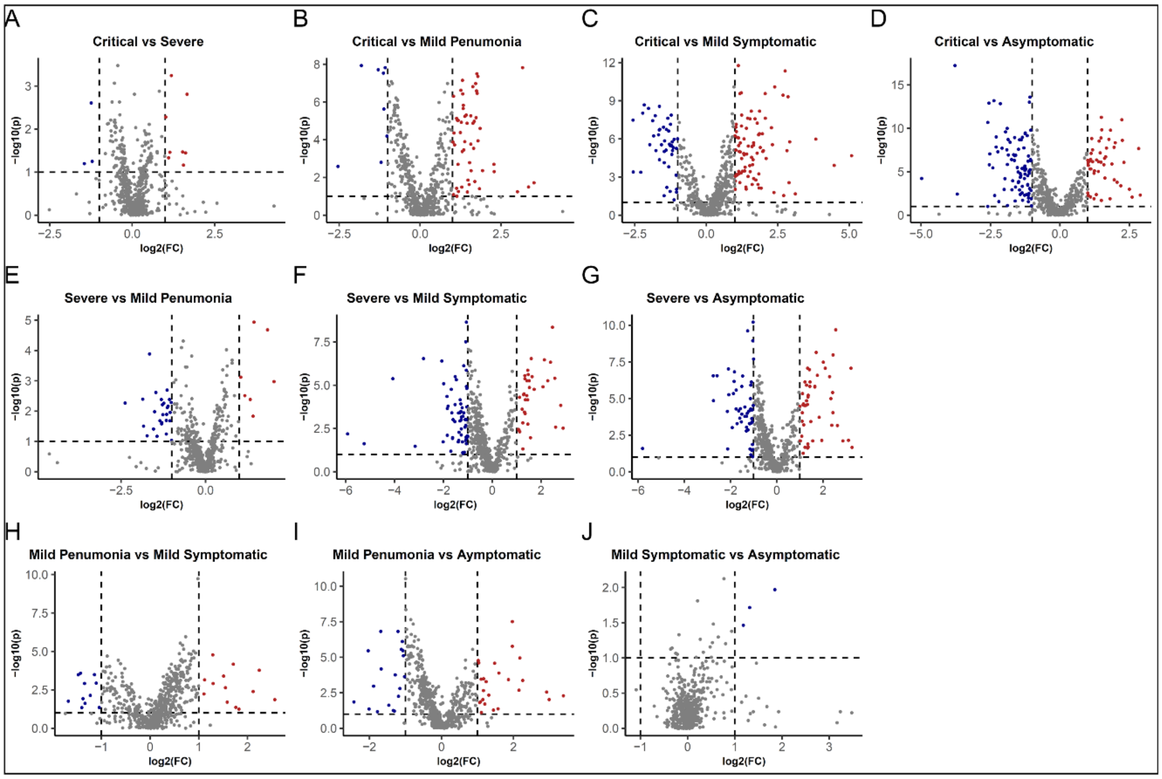

Supplementary Figure 1. Fold change volcano plots of metabolites associated with disease severity in COVID-19 patients. The x-axis represents the log2 fold change in metabolite levels between the two respective severity groups and the y-axis represents the -log10 p-value. Metabolites that are significantly associated with disease severity (p-value < 0.05 and fold change > 2) are represented by red dots. The dots in the base of the volcano represent the metabolites that were not found to be differentially expressed. The color code represents the direction of the fold change, red for upregulation and blue for downregulation.

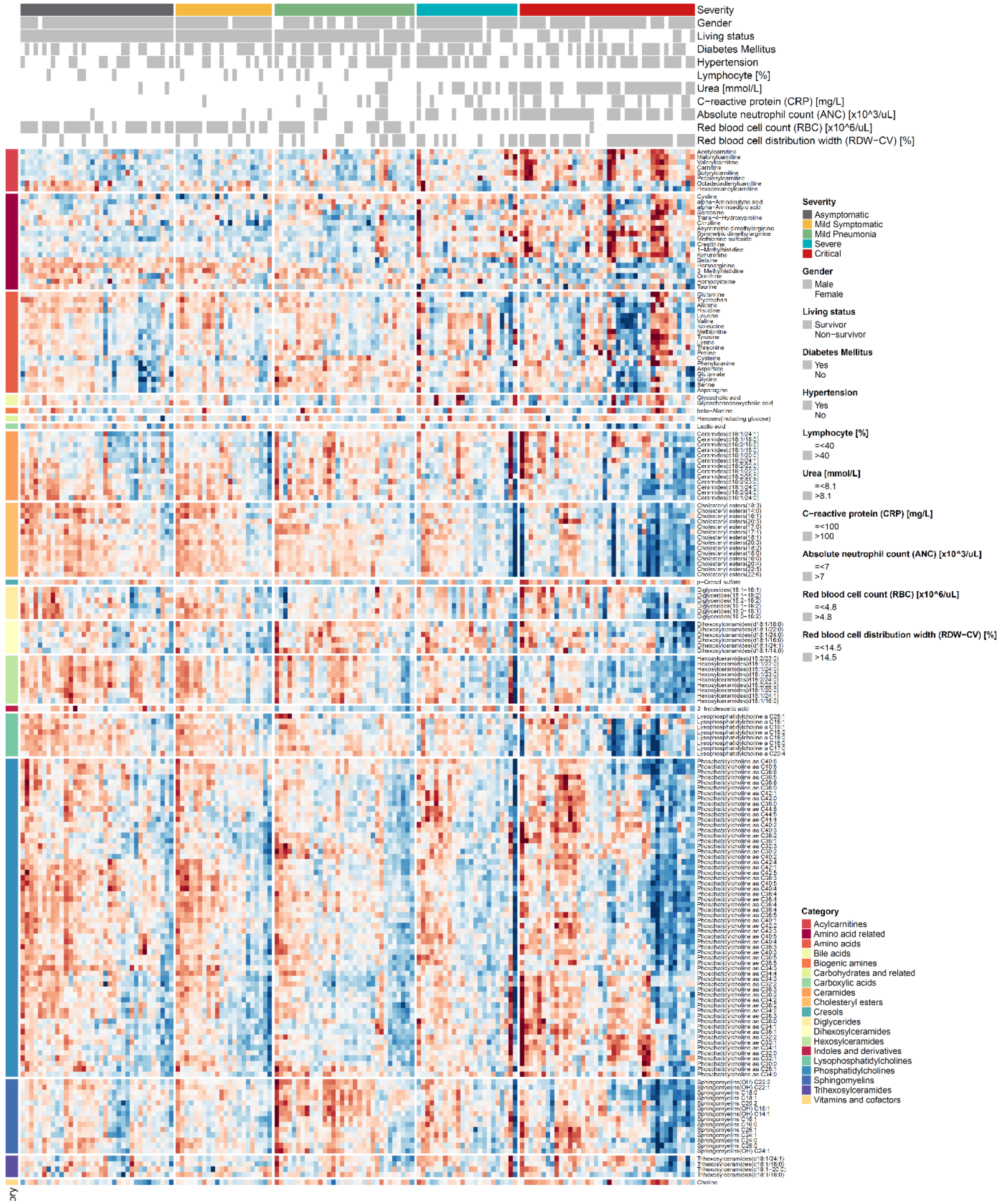

Supplementary Figure 2. Heatmap of several metabolite categories and clinical parameters associated with disease severity in COVID-19 patients. These categories includes lipids and related such as acylcarnitines, cholesteryl esters, diglycerides, dihexosylceramides, hexosylceramides, lysophosphatidylcholines, phosphatidylcholines, sphingomyelins, trihexosylceramides. Second, organic acids such as bile acids, carboxylic acids, and cresols. Third, amino acids and related: amino acid related, amino acids, biogenic amines, indoles and derivatives. Fourth, carbohydrates and vitamins such as carbohydrates and related, ceramides, vitamins and cofactors.

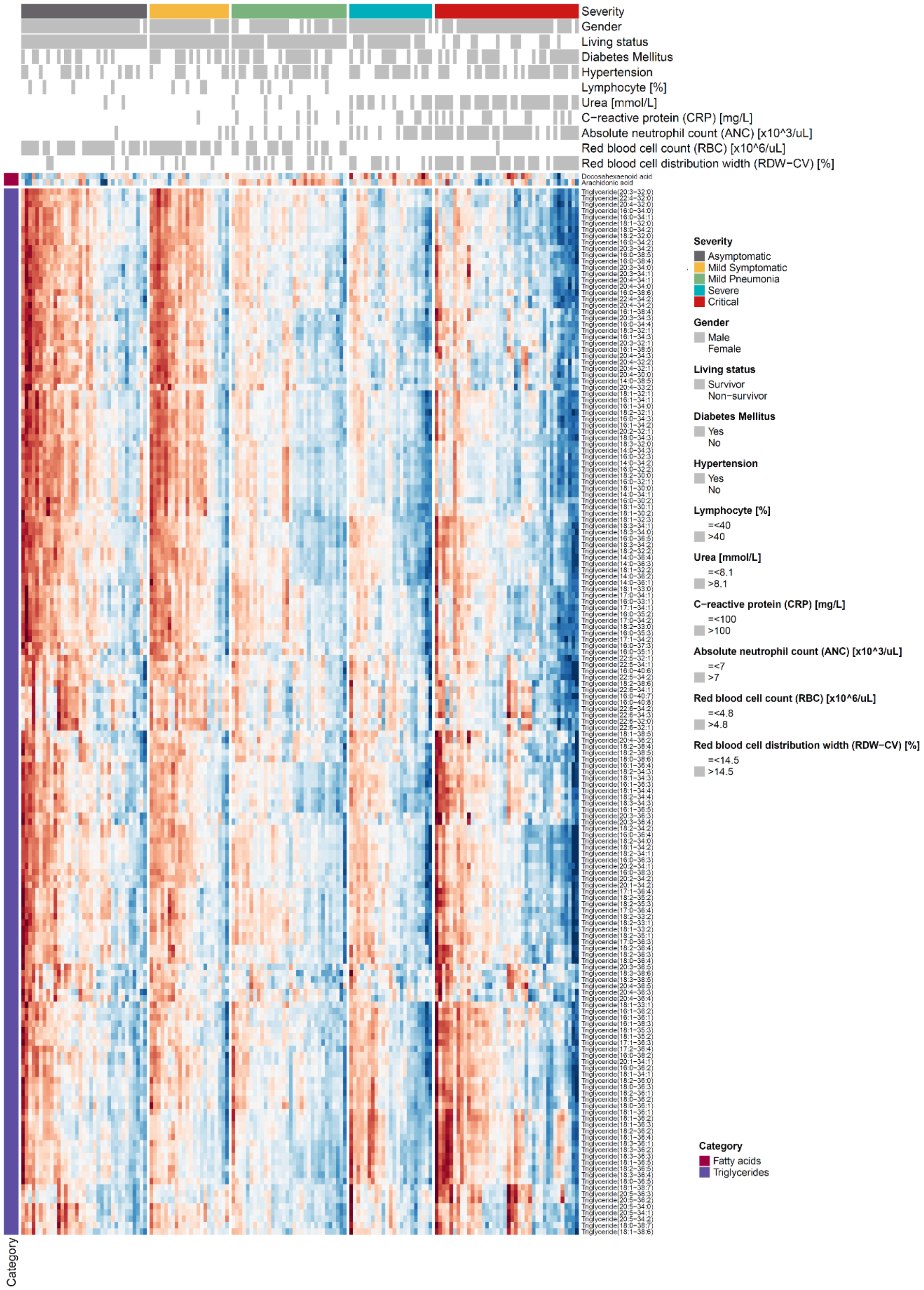

Supplementary Figure 3. Heatmap of fatty acids and triglycerides with clinical parameters data in COVID-19 patients with varying disease severity.
