# Supplementary material for: Metabolic predictors of COVID-19 mortality and severity: a survival analysis
Source: Front Immunol. 2024 May 10;15:1353903. doi: 10.3389/fimmu.2024.1353903 (PMC11127595; doi:10.3389/fimmu.2024.1353903)
Supplement: Supplementary file 2 [file DataSheet_2.pdf]

In [ ]:
```
import pandas as pd
import numpy as np
from sklearn.model_selection import train_test_split
from sklearn.linear_model import LogisticRegression
from sklearn.metrics import confusion_matrix,
accuracy_score
import itertools

import random

# Set random seed
random.seed(0)

# Load data into a pandas dataframe
df =
pd.read_csv("/Volumes/Samsung_T5/Science/metab_r



cols = ["Living
status","Alanine","Phenylalanine","Tryptophan",
"Serine","Cysteine","Glutamine", "Aspartate",
"Glutamate", "Histidine","Serotonin", "Tryptophan
betaine", "Kynurenine", "Acetylcarnitine",
"Propionylcarnitine", "Carnitine",
"Valerylcarnitine","Butyrylcarnitine", "Symmetric
dimethylarginine","Asymmetric dimethylarginine", "1-
Methylhistidine", "3-Methylhistidine"]
df = df[cols]
df.dropna(inplace=True)



# List of predictors
predictors = ["Tryptophan", "Kynurenine",
"Asymmetric dimethylarginine", "1-Methylhistidine"]
#"3-Methylhistidine"

# Split the data into training and testing sets
X_train, X_test, y_train, y_test =
train_test_split(df[predictors], df["Living status"],
test_size=0.2,random_state=20)
```

In [ ]:
```
# Export X_train, X_test, y_train, and y_test to CSV
X_train.to_csv('/Volumes/Samsung_T5/Science/metab_
 index=False)
X_test.to_csv('/Volumes/Samsung_T5/Science/metab_
 index=False)
y_train.to_csv('/Volumes/Samsung_T5/Science/metab_
 index=False)
y_test.to_csv('/Volumes/Samsung_T5/Science/metab_r
 index=False)
```

In [ ]:
```
X_train
```

	Tryptophan	Kynurenine	Asymmetric dimethylarginine	1-Methylhistidine
**118**	52.8	2.27	0.466	5.15

**0**	51.4	3.27	1.080	4.64
**48**	59.3	11.90	1.300	18.00
**11**	46.0	2.40	0.787	2.12
**109**	26.6	1.61	0.570	3.53
**...**	...	...	...	...
**76**	57.3	2.54	0.581	7.31
**150**	91.8	2.32	0.727	4.44
**139**	23.1	3.86	0.941	17.30
**15**	40.4	1.60	0.544	3.27
**100**	21.6	13.40	0.810	18.60

121 rows × 4 columns

In [ ]:X_test

	Tryptophan	Kynurenine	Asymmetric dimethylarginine	1-Methylhistidine
**113**	69.6	3.75	0.654	4.65
**67**	33.3	3.25	0.824	8.40
**107**	92.5	12.00	1.880	16.20
**131**	59.2	2.13	0.614	4.23
**135**	49.0	1.97	0.853	2.72
**92**	43.6	3.08	0.574	3.77
**123**	51.1	2.03	0.466	3.09
**21**	37.0	2.81	0.522	4.16
**85**	44.2	6.76	0.891	5.02
**12**	60.7	2.76	0.713	4.12
**140**	43.5	3.56	0.393	4.31
**125**	56.2	3.25	0.640	4.21
**75**	70.9	2.83	0.588	4.21
**36**	38.7	3.01	0.520	3.67
**29**	43.1	1.96	0.678	3.37
**88**	48.2	2.33	0.564	2.91

**13**	41.0	1.26	0.502	2.94
**134**	35.9	2.01	0.922	4.26
**122**	57.0	8.73	0.631	9.01
**63**	80.9	4.01	0.580	4.38
**74**	48.9	2.31	0.623	3.91
**141**	55.6	2.28	0.367	3.27
**114**	36.4	3.16	0.363	2.91
**20**	35.9	3.41	0.449	5.62
**57**	65.5	3.46	0.335	3.20
**133**	16.8	1.95	0.874	7.79
**101**	16.7	5.78	0.509	10.10
**35**	45.3	6.59	0.855	17.60
**96**	82.8	2.65	0.557	2.72
**86**	27.7	13.30	1.310	34.60
**98**	20.4	1.54	0.455	6.52

Random Forest Classifier

In [ ]:
```
from sklearn.ensemble import RandomForestClassifier
from sklearn.model_selection import train_test_split
from sklearn.metrics import accuracy_score, confusion_matrix
import pandas as pd

# Train Random Forest Classifier
rf_model = RandomForestClassifier(n_estimators=100, random_state=42)
rf_model.fit(X_train, y_train)

# Make predictions
y_pred = rf_model.predict(X_test)

# Evaluate model
accuracy = accuracy_score(y_test, y_pred)
conf_matrix = confusion_matrix(y_test, y_pred)

print("Random Forest Classifier")
print("Accuracy:", accuracy)
print("Confusion Matrix:\n", conf_matrix)

import matplotlib.pyplot as plt
import numpy as np
```

```
cm = np.array(confusion_matrix(y_test, y_pred))

plt.imshow(cm, interpolation='nearest',
cmap=plt.cm.Blues)
plt.title('Confusion Matrix')
plt.colorbar()

tick_marks = np.arange(2)
plt.xticks(tick_marks, [0, 1], rotation=45)
plt.yticks(tick_marks, [0, 1])

for i in range(2):
    for j in range(2):
        plt.text(j, i, format(cm[i, j], 'd'),
horizontalalignment="center", color="white" if cm[i,
j] > cm.max() / 2 else "black")

plt.tight_layout()
plt.ylabel('True living status')
plt.xlabel('Predicted living status')
plt.show()
```

```
Random Forest Classifier
Accuracy: 0.967741935483871
Confusion Matrix:
 [[26  1]
 [ 0  4]]
```

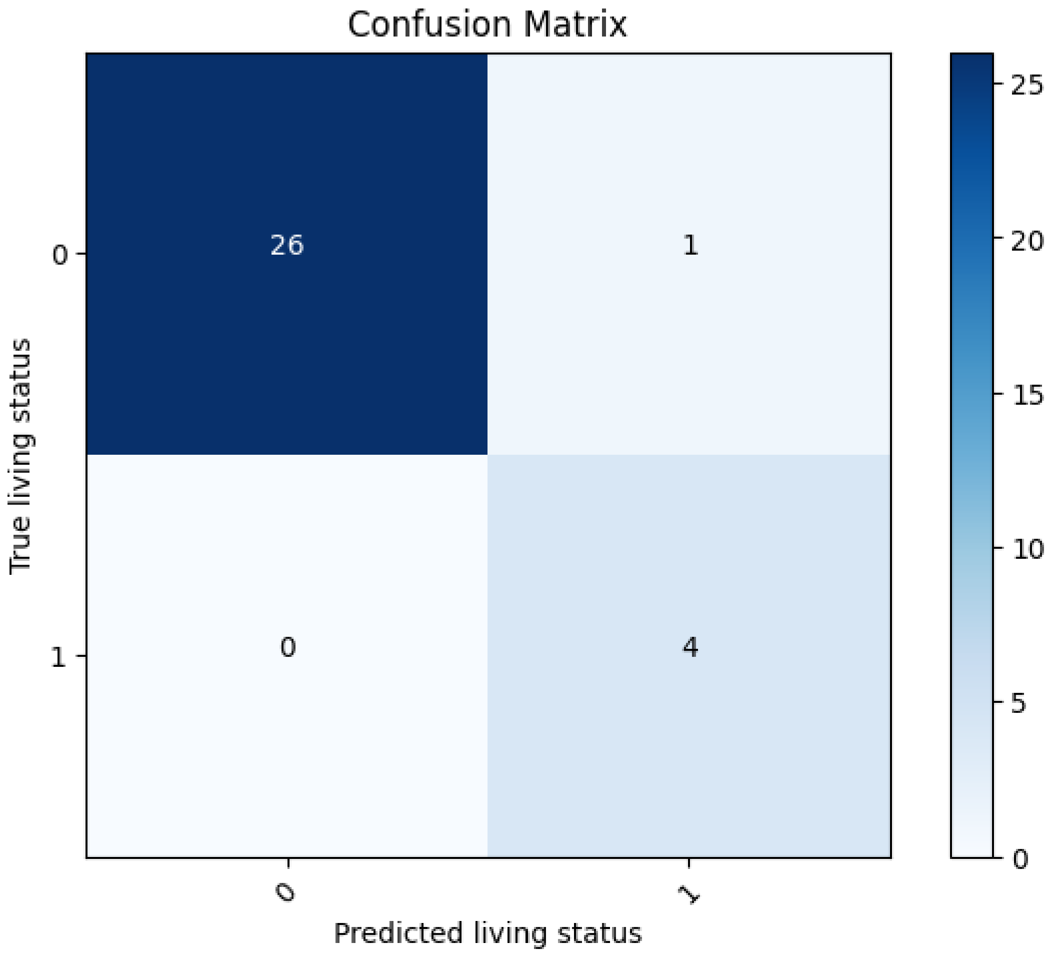

Support Vector Machine (SVM)

In [ ]:
```
from sklearn.svm import SVC
from sklearn.model_selection import train_test_split
from sklearn.metrics import accuracy_score,
confusion_matrix
import pandas as pd

# Train Support Vector Machine (SVM)
svm_model = SVC(kernel='rbf', random_state=42)
svm_model.fit(X_train, y_train)

# Make predictions
y_pred = svm_model.predict(X_test)

# Evaluate model
accuracy = accuracy_score(y_test, y_pred)
conf_matrix = confusion_matrix(y_test, y_pred)

print("Support Vector Machine (SVM)")
print("Accuracy:", accuracy)
print("Confusion Matrix:\n", conf_matrix)

import matplotlib.pyplot as plt
import numpy as np

cm = np.array(confusion_matrix(y_test, y_pred))

plt.imshow(cm, interpolation='nearest',
cmap=plt.cm.Blues)
plt.title('Confusion Matrix')
plt.colorbar()

tick_marks = np.arange(2)
plt.xticks(tick_marks, [0, 1], rotation=45)
plt.yticks(tick_marks, [0, 1])

for i in range(2):
    for j in range(2):
        plt.text(j, i, format(cm[i, j], 'd'),
horizontalalignment="center", color="white" if cm[i,
j] > cm.max() / 2 else "black")

plt.tight_layout()
plt.ylabel('True living status')
plt.xlabel('Predicted living status')
plt.show()
```

```
Support Vector Machine (SVM)
Accuracy: 0.8709677419354839
Confusion Matrix:
 [[26  1]
 [ 3  1]]
```

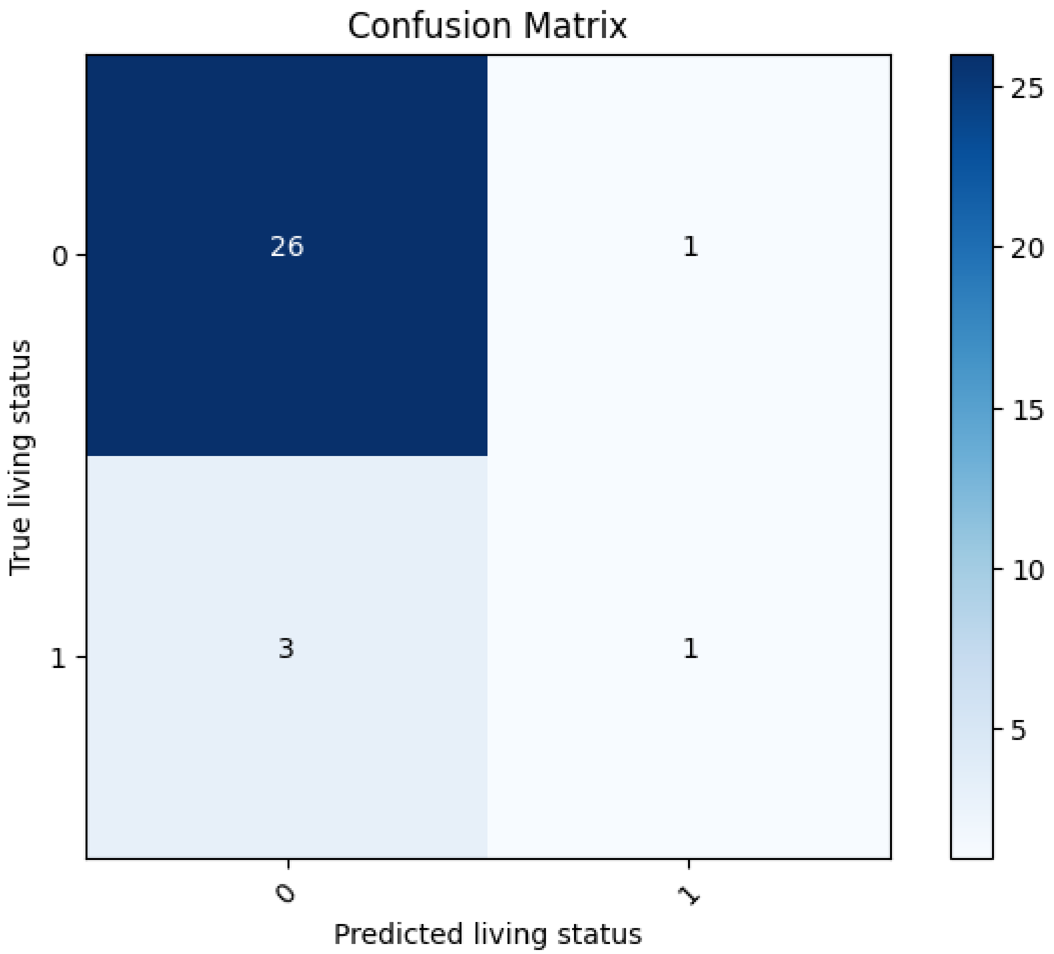

Bernoulli Naive Bayes

In [ ]:
```
from sklearn.naive_bayes import BernoulliNB
from sklearn.model_selection import train_test_split
from sklearn.metrics import accuracy_score, confusion_matrix
import pandas as pd

# Train Bernoulli Naive Bayes
nb_model = BernoulliNB()
nb_model.fit(X_train, y_train)

# Make predictions
y_pred = nb_model.predict(X_test)

# Evaluate model
accuracy = accuracy_score(y_test, y_pred)
conf_matrix = confusion_matrix(y_test, y_pred)

print("Bernoulli Naive Bayes")
print("Accuracy:", accuracy)
print("Confusion Matrix:\n", conf_matrix)

import matplotlib.pyplot as plt
import numpy as np

cm = np.array(confusion_matrix(y_test, y_pred))
```

```
plt.imshow(cm, interpolation='nearest',
cmap=plt.cm.Blues)
plt.title('Confusion Matrix')
plt.colorbar()

tick_marks = np.arange(2)
plt.xticks(tick_marks, [0, 1], rotation=45)
plt.yticks(tick_marks, [0, 1])

for i in range(2):
    for j in range(2):
        plt.text(j, i, format(cm[i, j], 'd'),
horizontalalignment="center", color="white" if cm[i,
j] > cm.max() / 2 else "black")

plt.tight_layout()
plt.ylabel('True living status')
plt.xlabel('Predicted living status')
plt.show()
```

```
Bernoulli Naive Bayes
Accuracy: 0.8709677419354839
Confusion Matrix:
 [[27  0]
 [ 4  0]]
```

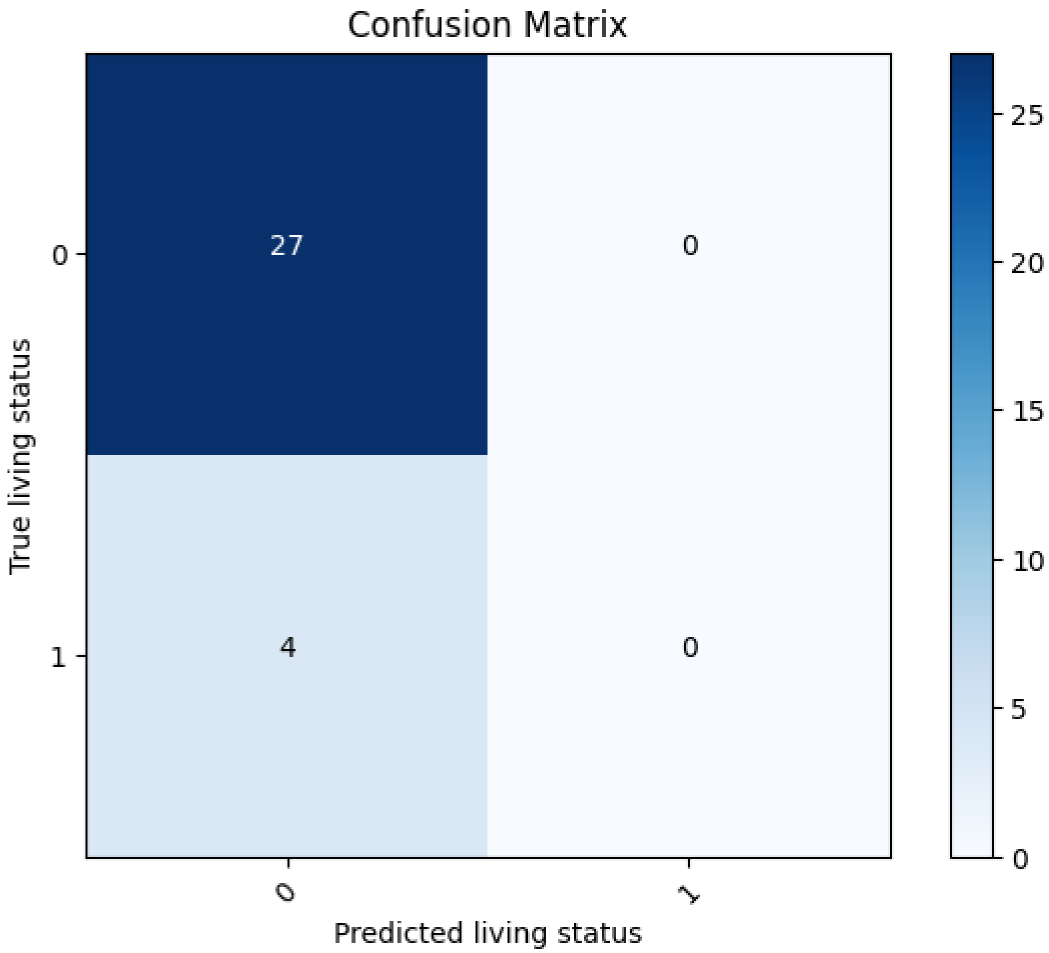

Gradient Boosting Classifier (using XGBoost)

In [ ]:
```
import xgboost as xgb
from sklearn.model_selection import train_test_split
```

```
from sklearn.metrics import accuracy_score,
confusion_matrix
import pandas as pd
from sklearn.preprocessing import LabelEncoder

# Encode categorical labels in y_train and y_test
label_encoder = LabelEncoder()

y_train = label_encoder.fit_transform(y_train)
y_test = label_encoder.transform(y_test)

# Train Gradient Boosting Classifier (XGBoost)
xgb_model = xgb.XGBClassifier()
xgb_model.fit(X_train, y_train)

# Make predictions
y_pred = xgb_model.predict(X_test)

# Evaluate model
accuracy = accuracy_score(y_test, y_pred)
conf_matrix = confusion_matrix(y_test, y_pred)

print("Gradient Boosting Classifier (XGBoost)")
print("Accuracy:", accuracy)
print("Confusion Matrix:\n", conf_matrix)

import matplotlib.pyplot as plt
import numpy as np

cm = np.array(confusion_matrix(y_test, y_pred))

plt.imshow(cm, interpolation='nearest',
cmap=plt.cm.Blues)
plt.title('Confusion Matrix')
plt.colorbar()

tick_marks = np.arange(2)
plt.xticks(tick_marks, [0, 1], rotation=45)
plt.yticks(tick_marks, [0, 1])

for i in range(2):
    for j in range(2):
        plt.text(j, i, format(cm[i, j], 'd'),
horizontalalignment="center", color="white" if cm[i,
j] > cm.max() / 2 else "black")

plt.tight_layout()
plt.ylabel('True living status')
plt.xlabel('Predicted living status')
plt.show()
```

```
Gradient Boosting Classifier (XGBoost)
Accuracy: 1.0
Confusion Matrix:
 [[27  0]
 [ 0  4]]
```

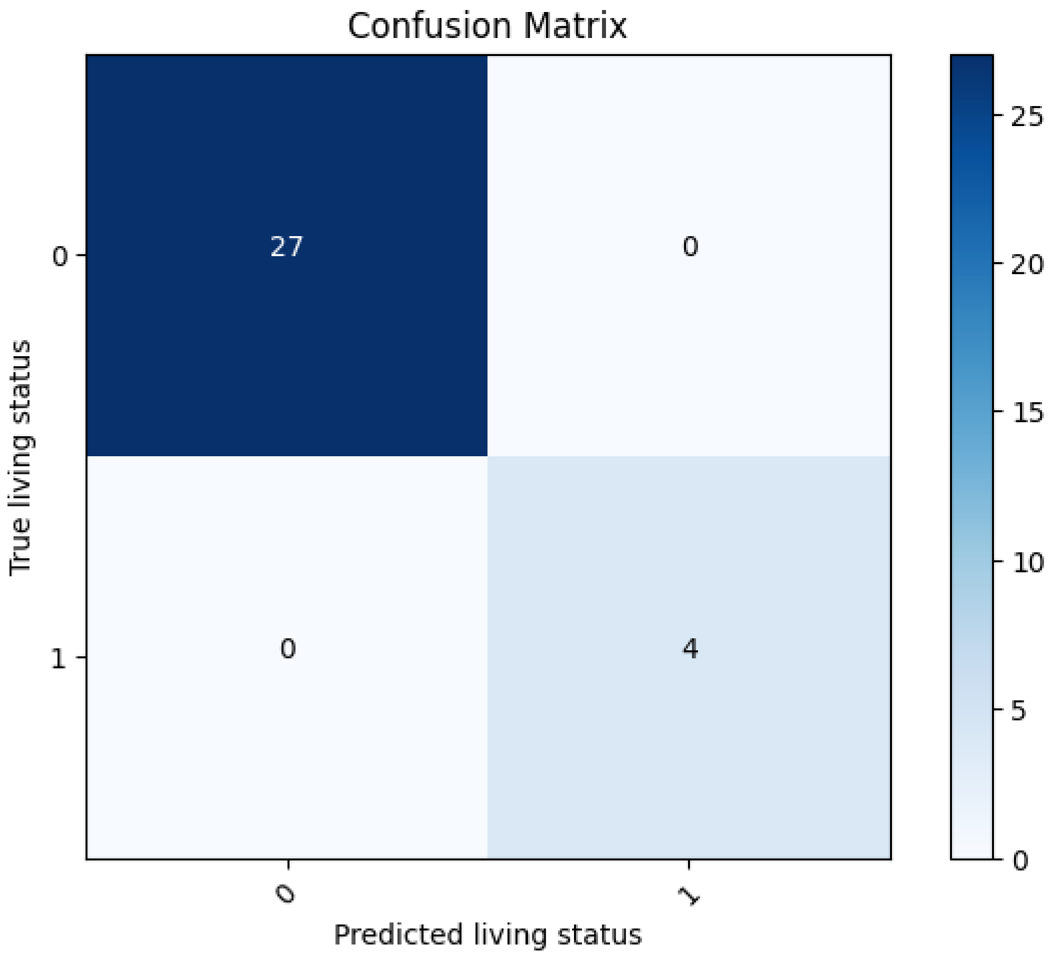

K-Nearest Neighbors (KNN):

In [ ]:
```
from sklearn.neighbors import KNeighborsClassifier
from sklearn.model_selection import train_test_split
from sklearn.metrics import accuracy_score,
confusion_matrix
import pandas as pd

# Train K-Nearest Neighbors (KNN)
knn_model = KNeighborsClassifier(n_neighbors=5)
knn_model.fit(X_train, y_train)

# Make predictions
y_pred = knn_model.predict(X_test)

# Evaluate model
accuracy = accuracy_score(y_test, y_pred)
conf_matrix = confusion_matrix(y_test, y_pred)

print("K-Nearest Neighbors (KNN)")
print("Accuracy:", accuracy)
print("Confusion Matrix:\n", conf_matrix)

import matplotlib.pyplot as plt
import numpy as np

cm = np.array(confusion_matrix(y_test, y_pred))
```

```
plt.imshow(cm, interpolation='nearest',
cmap=plt.cm.Blues)
plt.title('Confusion Matrix')
plt.colorbar()

tick_marks = np.arange(2)
plt.xticks(tick_marks, [0, 1], rotation=45)
plt.yticks(tick_marks, [0, 1])

for i in range(2):
    for j in range(2):
        plt.text(j, i, format(cm[i, j], 'd'),
horizontalalignment="center", color="white" if cm[i,
j] > cm.max() / 2 else "black")

plt.tight_layout()
plt.ylabel('True living status')
plt.xlabel('Predicted living status')
plt.show()
```

```
K-Nearest Neighbors (KNN)
Accuracy: 0.8709677419354839
Confusion Matrix:
 [[24  3]
 [ 1  3]]
```

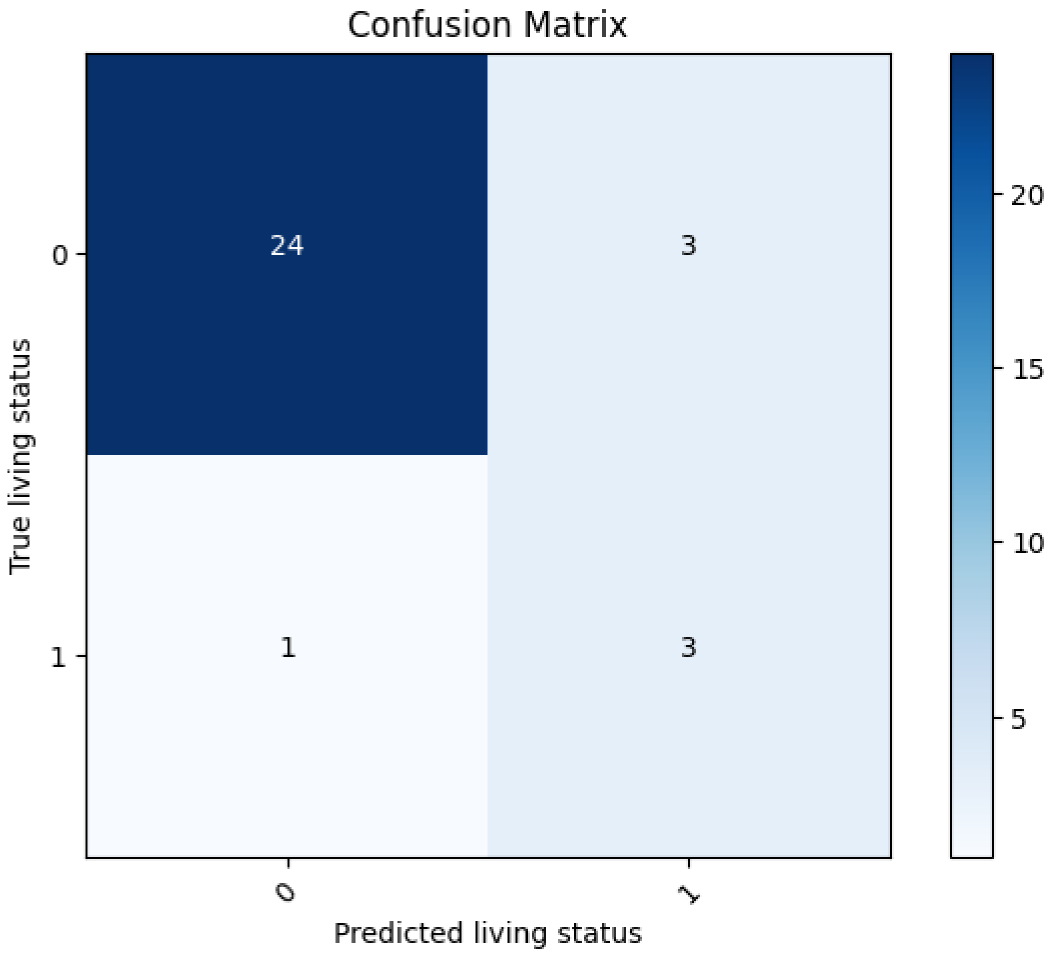

Neural Network (Multilayer Perceptron):

In [ ]:
```
from sklearn.neural_network import MLPClassifier
from sklearn.model_selection import train_test_split
```

```python
from sklearn.metrics import accuracy_score,
confusion_matrix
import pandas as pd

# Train Neural Network (Multilayer Perceptron)
mlp_model = MLPClassifier(hidden_layer_sizes=(100,
50), max_iter=500, random_state=42)
mlp_model.fit(X_train, y_train)

# Make predictions
y_pred = mlp_model.predict(X_test)

# Evaluate model
accuracy = accuracy_score(y_test, y_pred)
conf_matrix = confusion_matrix(y_test, y_pred)

print("Neural Network (Multilayer Perceptron)")
print("Accuracy:", accuracy)
print("Confusion Matrix:\n", conf_matrix)

import matplotlib.pyplot as plt
import numpy as np

cm = np.array(confusion_matrix(y_test, y_pred))

plt.imshow(cm, interpolation='nearest',
cmap=plt.cm.Blues)
plt.title('Confusion Matrix')
plt.colorbar()

tick_marks = np.arange(2)
plt.xticks(tick_marks, [0, 1], rotation=45)
plt.yticks(tick_marks, [0, 1])

for i in range(2):
    for j in range(2):
        plt.text(j, i, format(cm[i, j], 'd'),
horizontalalignment="center", color="white" if cm[i,
j] > cm.max() / 2 else "black")

plt.tight_layout()
plt.ylabel('True living status')
plt.xlabel('Predicted living status')
plt.show()
```

```
Neural Network (Multilayer Perceptron)
Accuracy: 0.9032258064516129
Confusion Matrix:
 [[25  2]
 [ 1  3]]
/opt/anaconda3/envs/freepik/lib/python3.12/site-packages/sklearn/neural_network/_multilayer_perceptron.p
y:691: ConvergenceWarning: Stochastic Optimizer: Maximum iterations (500) reached and the optimization h
asn't converged yet.
  warnings.warn(
```

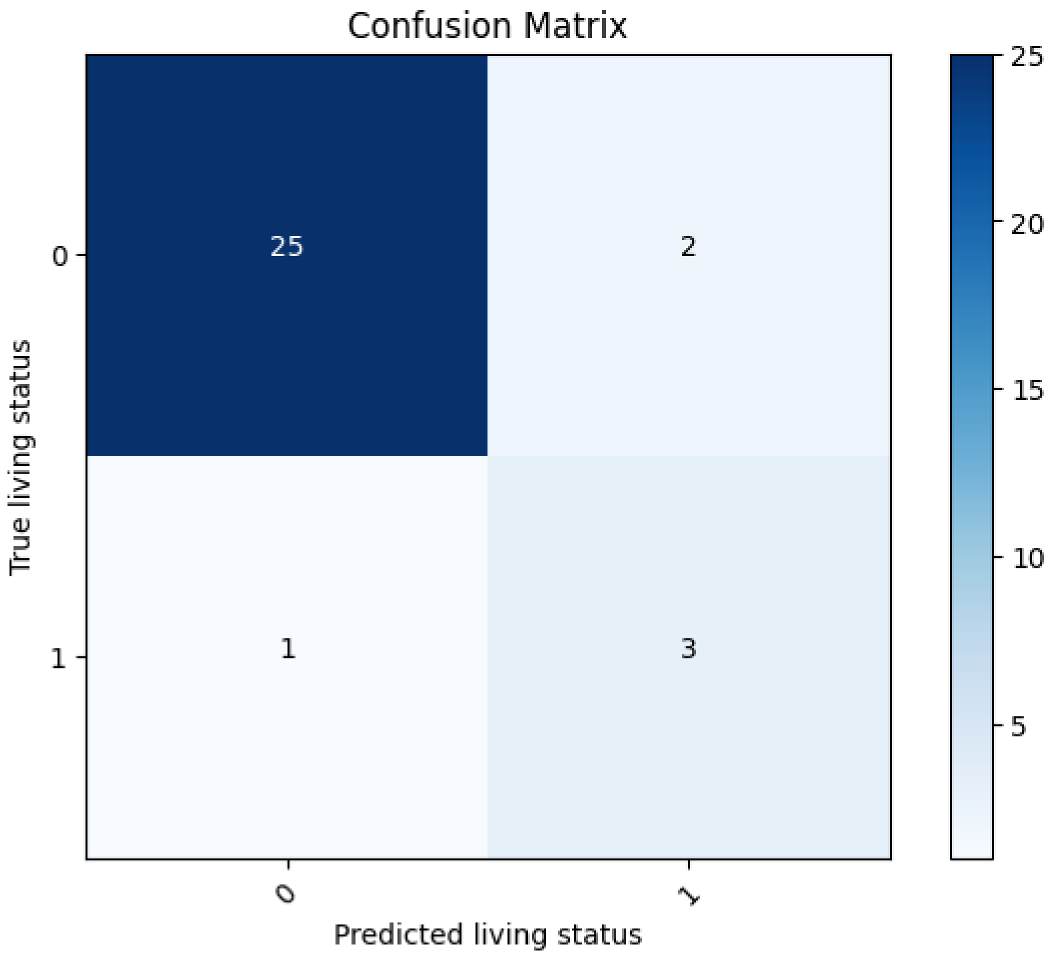

Logistic Regression

In [ ]:
```
from sklearn.linear_model import LogisticRegression
from sklearn.model_selection import train_test_split
from sklearn.metrics import accuracy_score,
confusion_matrix
import pandas as pd

# Train Logistic Regression
log_reg_model = LogisticRegression()
log_reg_model.fit(X_train, y_train)

# Make predictions
y_pred = log_reg_model.predict(X_test)

# Evaluate model
accuracy = accuracy_score(y_test, y_pred)
conf_matrix = confusion_matrix(y_test, y_pred)

print("Logistic Regression")
print("Accuracy:", accuracy)
print("Confusion Matrix:\n", conf_matrix)

import matplotlib.pyplot as plt
import numpy as np

cm = np.array(confusion_matrix(y_test, y_pred))
```

```
plt.imshow(cm, interpolation='nearest',
cmap=plt.cm.Blues)
plt.title('Confusion Matrix')
plt.colorbar()

tick_marks = np.arange(2)
plt.xticks(tick_marks, [0, 1], rotation=45)
plt.yticks(tick_marks, [0, 1])

for i in range(2):
    for j in range(2):
        plt.text(j, i, format(cm[i, j], 'd'),
horizontalalignment="center", color="white" if cm[i,
j] > cm.max() / 2 else "black")

plt.tight_layout()
plt.ylabel('True living status')
plt.xlabel('Predicted living status')
plt.show()
```

```
Logistic Regression
Accuracy: 0.9354838709677419
Confusion Matrix:
 [[26  1]
 [ 1  3]]
```

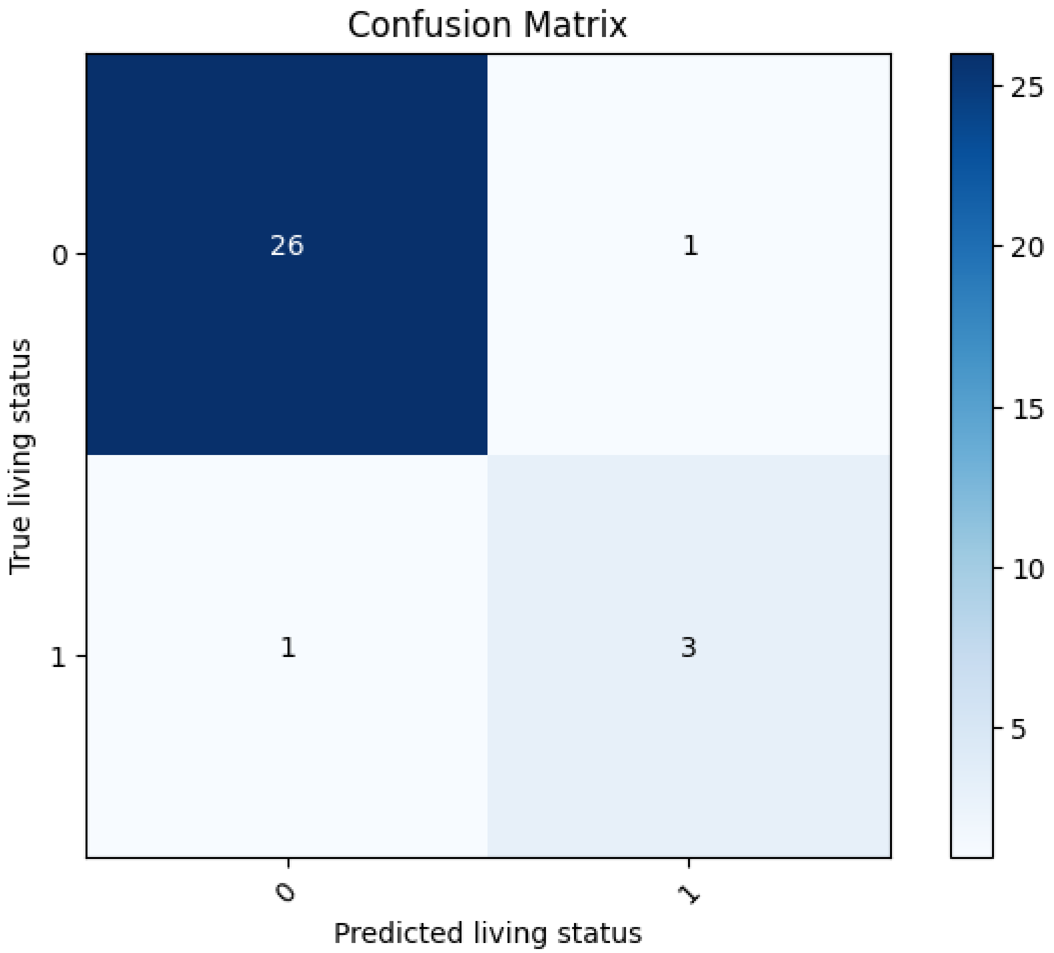

MLPClassifier

In [ ]:
```
from sklearn.neural_network import MLPClassifier
from sklearn.model_selection import train_test_split
```

```python
from sklearn.metrics import accuracy_score, confusion_matrix
import pandas as pd

# Train MLPClassifier (Multilayer Perceptron)
mlp_model = MLPClassifier(hidden_layer_sizes=(100, 50), max_iter=1000, random_state=42)
mlp_model.fit(X_train, y_train)

# Make predictions
y_pred = mlp_model.predict(X_test)

# Evaluate model
accuracy = accuracy_score(y_test, y_pred)
conf_matrix = confusion_matrix(y_test, y_pred)

print("MLPClassifier (Multilayer Perceptron)")
print("Accuracy:", accuracy)
print("Confusion Matrix:\n", conf_matrix)

import matplotlib.pyplot as plt
import numpy as np

cm = np.array(confusion_matrix(y_test, y_pred))

plt.imshow(cm, interpolation='nearest', cmap=plt.cm.Blues)
plt.title('Confusion Matrix')
plt.colorbar()

tick_marks = np.arange(2)
plt.xticks(tick_marks, [0, 1], rotation=45)
plt.yticks(tick_marks, [0, 1])

for i in range(2):
    for j in range(2):
        plt.text(j, i, format(cm[i, j], 'd'), horizontalalignment="center", color="white" if cm[i, j] > cm.max() / 2 else "black")

plt.tight_layout()
plt.ylabel('True living status')
plt.xlabel('Predicted living status')
plt.show()
```

```
MLPClassifier (Multilayer Perceptron)
Accuracy: 0.9032258064516129
Confusion Matrix:
 [[24  3]
 [ 0  4]]
```

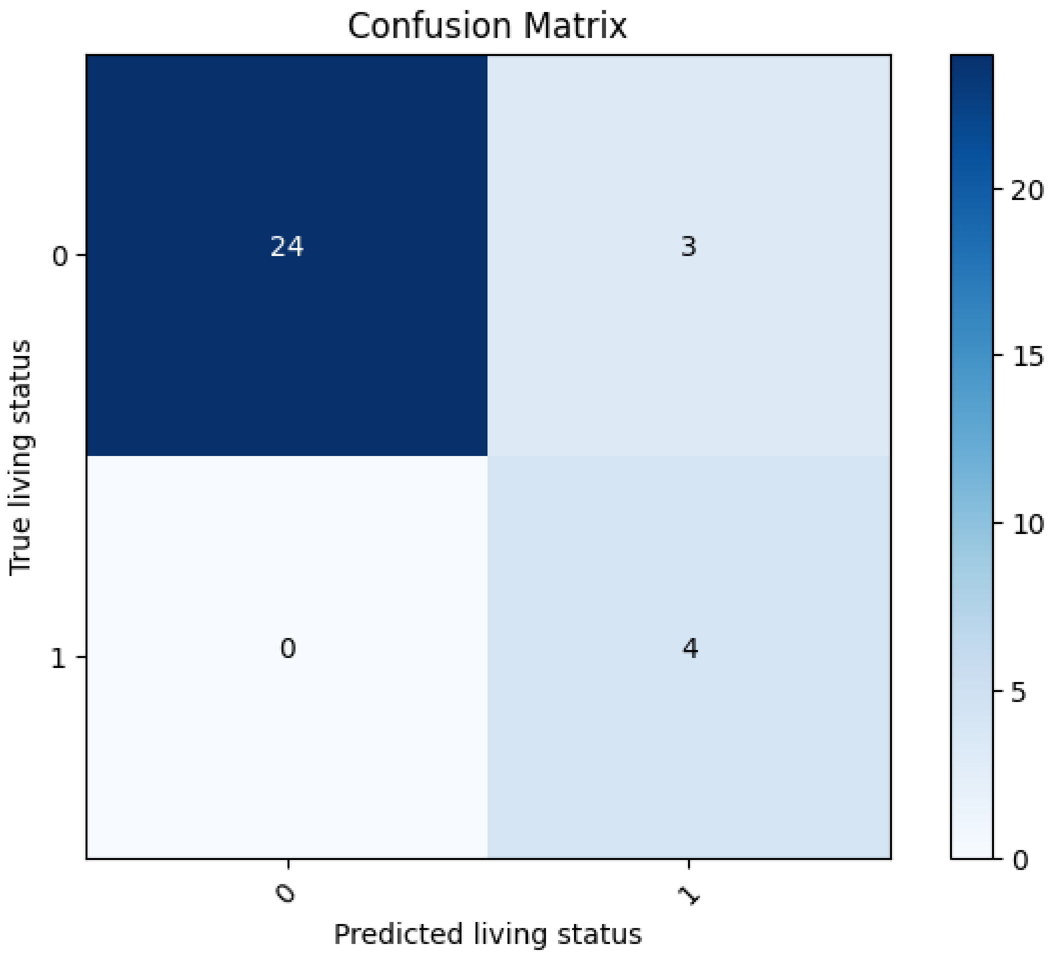
Confusion Matrix
True living status
Predicted living status
24
3
0
4
0
1
0
1
20
15
10
5
0
